# NF-κB Activator 1 downregulation in macrophages activates STAT3 to promote adenoma-adenocarcinoma transition and immunosuppression in colorectal cancer

**DOI:** 10.1186/s12916-023-02791-0

**Published:** 2023-03-29

**Authors:** Shunyi Wang, Yihe Kuai, Simin Lin, Li Li, Quliang Gu, Xiaohan Zhang, Xiaoming Li, Yajun He, Sishuo Chen, Xiaoru Xia, Zhang Ruan, Caixia Lin, Yi Ding, Qianqian Zhang, Cuiling Qi, Jiangchao Li, Xiaodong He, Janak L. Pathak, Weijie Zhou, Side Liu, Lijing Wang, Lingyun Zheng

**Affiliations:** 1 School of Life Science and Biopharmaceutics, Guangdong Pharmaceutical University, Guangzhou, 510006 Guangdong P. R. China; 2 Department of Gastroenterology, Guangdong Provincial Key Laboratory of Gastroenterology, Nanfang Hospital, Southern Medical University, Guangzhou, Guangdong China; 3 Hospital of Guangdong Provincial Hospital of Traditional Chinese Medicine, Zhuhai, 519015 China; 4 Department of Pathology, People’s Hospital of Shenzhen Bao an District, Shenzhen, 518101 China; 5 Guangdong Engineering Research Center of Oral Restoration and Reconstruction, Guangzhou Key Laboratory of Basic and Applied Research of Oral Regenerative Medicine, Affiliated Stomatology Hospital of Guangzhou Medical University, Guangdong 510182 Guangzhou, China; 6 Department of General Surgery & Guangdong Provincial Key Laboratory of Precision Medicine for Gastrointestinal Tumor, Nanfang Hospital, First Clinical Medical School, Southern Medical University, Guangzhou, 510515 P. R. China

**Keywords:** Act1, Macrophages, Colorectal cancer, Adenoma-adenocarcinoma transition, CD8^+^ T cells

## Abstract

**Background:**

Adenoma-adenocarcinoma transition is a key feature of colorectal cancer (CRC) occurrence and is closely regulated by tumor-associated macrophages (TAMs) and CD8^+^ T cells. Here, we investigated the effect of the NF-κB activator 1 (Act1) downregulation of macrophages in the adenoma-adenocarcinoma transition.

**Methods:**

This study used spontaneous adenoma-developing Apc^Min/+^, macrophage-specific Act1-knockdown (anti-Act1), and Apc^Min/+^; anti-Act1 (AA) mice. Histological analysis was performed on CRC tissues of patients and mice. CRC patients’ data retrieved from the TCGA dataset were analyzed. Primary cell isolation, co-culture system, RNA-seq, and fluorescence-activated cell sorting (FACS) were used.

**Results:**

By TCGA and TISIDB analysis, the downregulation of Act1 expression in tumor tissues of CRC patients negatively correlated with accumulated CD68^+^ macrophages in the tumor. Relative expression of EMT markers in the tumor enriched ACT1^low^CD68^+^ macrophages of CRC patients. AA mice showed adenoma-adenocarcinoma transition, TAMs recruitment, and CD8^+^ T cell infiltration in the tumor. Macrophages depletion in AA mice reversed adenocarcinoma, reduced tumor amounts, and suppressed CD8^+^ T cell infiltration. Besides, macrophage depletion or anti-CD8a effectively inhibited metastatic nodules in the lung metastasis mouse model of anti-Act1 mice. CRC cells induced activation of IL-6/STAT3 and IFN-γ/NF-κB signaling and the expressions of CXCL9/10, IL-6, and PD-L1 in anti-Act1 macrophages. Anti-Act1 macrophages facilitated epithelial-mesenchymal-transition and CRC cells’ migration via CXCL9/10-CXCR3-axis. Furthermore, anti-Act1 macrophages promoted exhaustive PD1^+^ Tim3^+^ CD8^+^ T cell formation. Anti-PD-L1 treatment repressed adenoma-adenocarcinoma transition in AA mice. Silencing STAT3 in anti-Act1 macrophages reduced CXCL9/10 and PD-L1 expression and correspondingly inhibited epithelial-mesenchymal-transition and CRC cells’ migration.

**Conclusions:**

Act1 downregulation in macrophages activates STAT3 that promotes adenoma-adenocarcinoma transition via CXCL9/10-CXCR3-axis in CRC cells and PD-1/PD-L1-axis in CD8^+^ T cells.

**Supplementary Information:**

The online version contains supplementary material available at 10.1186/s12916-023-02791-0.

## Background

Colorectal cancer (CRC), one of the most deadly and common types of cancer [1], is mainly caused by slow progression from precancerous adenoma. A total of 60–90% of sporadic CRC arise via the classical adenoma-carcinoma pathway [2]. Advanced adenomas are the most frequent premalignant precursor lesions of sporadic CRC [3, 4]. The transition from tubular adenoma to adenocarcinoma occurs over a period of more than 10 to 15 years [5]. The loss of the adenomatous polyposis coli (APC) gene, a regulator of the Wnt/beta-catenin pathway, is considered the initial step of the adenoma-carcinoma sequence and leads to abnormal cell proliferation and the formation of adenomatous polyposis [6]. Accumulated evidence has identified that chronic inflammation plays a key role in CRC initiation, promotion, and progression [7]. However, which cells in the tumor microenvironment are responsible for classical adenoma-carcinoma pathway-mediated sporadic CRC is still unknown.

Inflammatory cells are key components of the tumor microenvironment, and macrophages represent the most abundant leukocyte population infiltrating neoplastic tissues [8–10]. Tumor-associated macrophages (TAMs) polarization and their cytokines regulate the progression and metastasis of CRC [11]. Similarly, polarized macrophages and cytokines such as IL-6, IFN-γ, IL-1, etc. play an indispensable role in the progression of adenomatous polyposis [12]. In addition, PD-L1 (Programmed Cell Death Ligand 1) and PD-L2 (Programmed Cell Death Ligand 2) from TAMs or tumor cells promote the inhibitory function of the PD-1(Programmed Cell Death Protein 1) immune checkpoint in T cells [13, 14]. However, the role of TAMs in the adenoma-adenocarcinoma transition during CRC development is still unclear. Among cytokine signaling pathways, myeloid cell-specific NF-κB signaling is a central axis of proinflammatory cytokine production affecting tumor growth in multiple cancer models [15, 16].

NF-κB activator 1 (Act1), an adaptor protein, is also known as a connection to IKK and SAPK/JNK (CIKS) and TRAF3-interacting protein 2 (TRAF3IP2) [17]. Act1 has been shown to mediate the activation of both NF-κB and AP-1 in an IKK- and JNK-dependent manner [18]. Act1-deficient mice exhibit less severe allergic airway inflammation, pulmonary inflammation, and dextran sodium sulfate-induced colitis [19–21], suggesting a causal role for Act1 in autoimmune and inflammatory disorders. Therefore, as an NF-κB activator, the Act1 level in macrophages is likely to modulate TAMs to regulate CRC development and progression.

This study aimed to analyze the role of downregulated Act1 in macrophages in adenoma-adenocarcinoma transition in CRC and the underlying mechanisms. Using Apc^Min/+^ mice (a spontaneous adenoma model) and macrophage-specific Act1 knockdown (anti-Act1) mice, we generated Apc^Min/+^; anti-Act1 (AA) mice. We first observed adenoma-adenocarcinoma transition accompanied by infiltration of TAMs and CD8^+^ T cells in tumor tissues and shortened the overall survival of AA mice. Meanwhile, macrophage depletion significantly inhibited adenocarcinoma transition, adenoma formation, and recruitment of CD8^+^ T cells in AA mice and in the lung metastasis model of anti-Act1 mice. Moreover, anti-CD8a treatment effectively reduced lung metastatic nodules in anti-Act1 mice. In vitro, CRC cells induced CXCL9/10, IL-6 secretion, and PD-L1 expression in anti-Act1 macrophages via STAT3 activation. Interestingly, anti-Act1 macrophages induced CXCR3 (CXCL9/10 receptor) expression in CRC cells to promote epithelial-mesenchymal transition and CRC cells’ migration. Act-1 knockdown macrophages also increased the number of exhaustive PD1^+^Tim3^+^CD8^+^ T cells after coculture with CRC cells. Importantly, the PD-L1 inhibitor effectively suppressed adenocarcinoma transition and reduced tumor amounts in AA mice. Our results showed that Act1 downregulation in macrophages activates STAT3 to promote adenoma-adenocarcinoma transition via CXCL9/10-CXCR3-axis in CRC cells and immunosuppression via PD-1/PD-L1-axis in CD8^+^ T cells.

## Methods

### Clinical specimens for pathological analysis

A total of 65 cases of tumor tissues of patients with colorectal cancer, with a median age of 66 years (ranging from 33 to 87 years), and their matched non-tumorous colorectal tissues, as well as the corresponding clinical data, were collected from the Department of Pathology, People’s Hospital of Baoan District (Shenzhen, China) and the Department of Gastroenterology, Nanfang Hospital (Guangzhou, China). Pathologic diagnosis was performed by two independent pathologists based on the guidelines of the International Union Against Cancer (UICC). All samples were collected with informed consent from patients, and all related procedures were performed with the approval of the internal review and ethics boards of People’s Hospital of Baoan District of Shenzhen (Approval Number: BYL20220401) and Nanfang Hospital. Additional file 1: Table S1 summarizes the CRC patients’ demographics and clinical information.

### Animal experiments

Male and female C57BL/6 J-Apc^Min/+^ mice were purchased from the Jackson Laboratory (Jackson Laboratories, Bar Harbor, ME). Anti-Act1 mice were developed and described previously [22]. Male Apc^Min/+^ mice were mated with female anti-Act1 mice on the C57BL/6 J background to obtain AA mice. Their genotypes were identified by PCR using the following primers: Apc^Min/+^, forward: 5′-GCCATCCCTTCACGTTAG-3′, 5′-TTCCACTTTGGCATAAGGC-3′; reverse: TTCTGAGAAAGACAGAAGTTA; and anti-Act1, forward: 5′-CTGGTGCAGACAGCCTAGCTG-3′; reverse: 5′-CCTGCGAGCTAAAGT CCTGGA-3. The PCR product was separated by 1.2% agarose gel and visualized using a gel imager (Model G-box EF Syngene, USA). The detail of phenotyping assays is shown in Additional file 2: Figure S1. Mice were fed a normal diet and maintained at the Laboratory Animal Center of Guangdong Pharmaceutical University. Nine-week, 12-week, and 15-week male Apc^Min/+^ and AA mice were used for pathological analysis. For xenograft models, a single cell suspension of the MC38 CRC cells (5 × 10^5^/ml) was subcutaneously implanted into the left lumbar region of adult anti-Act1 mice and C57BL/6 J mice. Primary tumors were dissected from the sacrificed mice after 2 weeks, and the tumors were harvested for immunohistochemistry. For in vivo anti-PD-L1 treatment models, 12 weeks old male AA mice were randomly divided into two groups and treated with sterile phosphate-buffered saline (PBS) or anti-PD-L1 antibody (10 mg/kg, #1,380,723–44-3, Selleck) by intraperitoneal injection 2 times per week for 3 weeks. After the mice were sacrificed, the intestines were excised from each mouse. The intestines were cut longitudinally, and luminal contents were washed out using PBS. After unfolding on filter papers, the intestines were fixed in 10% phosphate-buffered formalin overnight. The next day, the intestines were stained with 0.1% (v/v) methylene blue. The numbers and diameters of the tumors in the small and large intestines were measured using an inverted microscope, and tumor volumes were calculated with the following equation: volume = 0.52 ⨯ (length ⨯ width^2^). For the lung metastasis mouse model, the detailed methods are presented in Additional file 3: Supplementary Methods.

All experiments were carried out in accordance with the "Guidelines for the Care and Use of Laboratory Animals of Guangdong Pharmaceutical University" and were approved by the Experimental Animal Ethics Committee of Guangdong Pharmaceutical University (Approval Number: Gdpulac2017026).

### Histology and immunohistochemistry

Paraffin-embedded intestines were sectioned and stained with H&E. Two senior pathologists graded the stained slides and defined the adenomas as low or high grades according to the consensus recommendations [23]. For immunohistochemistry assays, 3 µm thick tissue sections were subsequently dewaxed, deparaffinized, and rehydrated, and endogenous peroxidase was quenched with 3% H_2_O_2_ in methanol for 30 min. The slides were blocked with 10% bovine serum albumin (Sigma-Aldrich) at 37 °C for 50 min and then incubated with anti-CD68 (1:100; #ab213363, Abcam, America), anti-F4/80 (1:200; #70076 s, CST, America), anti-Ki67 (1:200; # ab15580, Abcam, America), anti-CD8 (1:200; #ab217344, Abcam, America), anti-E-cadherin (1:400, #3195, CST, America), anti-Snail (1:100, #A5243, ABclonal, China), anti-CXCR3 (1:100; #abs136633, Absin, China), anti-β-catenin (1:100, #610154, BD Biosciences, America), anti-CA19-9 (1:100, #ab15146, Abcam, America), and anti-Act-1 (1:50; #sc-100647, Santa Cruz Biotechnology, America) monoclonal antibodies were incubated overnight at 4 °C. Next, the tissue sections were incubated with a horseradish peroxidase-conjugated goat anti-rabbit or anti-mouse IgG antibody (ZSGB-BIO, Beijing, China) at 37 °C for 50 min. The slides were stained with diaminobenzidine solution (Dako Cytomation, Hamburg, Germany), and the cell nuclei were counterstained with hematoxylin. The images were taken with a microscope (OLYMPUS, Japan) 20 × field or 40 × field. The immunohistochemical staining data were collected and assessed quantified using Image-Pro Plus 4.5 software (Media Cybernetics, MD, USA).by two researchers using a double-blind protocol. For the lung metastasis mouse model, the detailed histological and immunohistochemistry assay are presented in Additional file 3: Supplementary methods.

### Immunofluorescent staining

To observe F4/80^+^ PD-L1^+^/CD68^+^ PD-L1^+^ macrophage and CD8^+^ PD1^+^ T cells in CRC tissue of patients and AA mice, the tissue slides were de-paraffinized, rehydrated, incubated for 30 min in sodium citrate buffer (10 mM sodium citrate, pH 6.0, 0.05% Tween-20) for antigen retrieval. The tissue was then blocked with a buffer containing 3% bovine serum albumin and 5% normal serum from the host species of the secondary antibody. Subsequently, sections were stained for 90 min at room temperature with the following primary antibodies in antibody diluent immunofluorescent staining was performed using the following antibodies: for mouse tissues, anti-F4/80 (mouse, 1:100; #30325 T, CST, America), anti-PD-L1(mouse, 1:100; #NBP1-76769, Novus, America), anti-CD8 (mouse, 1:100; #NBP1-49045, Novus, America), anti-PD-1(mouse, 1:100; #ab214421, Abcam, America), and matching Alexa Fluor (AF) 488- or AF594-coupled secondary antibodies (1:200, #A-11008, #A32758, #A-11006, #A-11012, Life Technologies) were used. Cell nuclei were counterstained with 4'-6-diamidino-2-phenylindole (DAPI). Sections were observed with a ZEISS LSM880 laser confocal microscope (Carl Zeiss, Jena, Germany) and the images were captured with ZEISS ZEN Microscopy software under a 100 × field.

For human tissues, anti-Act-1 (1:50; #sc-100647, Santa, America), anti-CD68 (human, 1:100; ab213363, Abcam, America), anti-PD-L1 (human, 1:100; #ab205921, Abcam, America), anti-CD8 (human, 1:100; #ab245118, Abcam, America), anti-PD-1(human, 1:100; #ab52587, Abcam, America), and matching Alexa Fluor (AF) 488- or AF594-coupled secondary antibodies (1:200, #A-11008, #A-11012, #A-11001, Life Technologies) were used. Cell nuclei were counterstained with 4'-6-diamidino-2-phenylindole (DAPI). Sections were observed with an Olympus DP70 fluorescence microscope (Olympus Corp., Tokyo, Japan) and the images were captured with DPControler software under a 40 × or 100 × field.

### Cell line culture and siRNA transfection

The two CRC cell lines MC38 and CT26 obtained from the Department of Pathology of Southern Medical University were routinely cultured in RPMI 1640 medium (Gibco) supplemented with 10% FBS (Gibco) and 1% penicillin–streptomycin (Sigma). All cells were authenticated and tested for Mycoplasma. Cells were cultured and allowed to grow as a monolayer in 5% CO_2_ and 37 °C and then collected with trypsin solution (0.5% w/v in PBS). For siRNA transfection, we used Lipofectamine 3000 (#L3000015, Thermo Scientific, USA) in 6-well tissue culture plates. Briefly, 100 nM of synthesized siRNA sequences for CXCR3 (RiboBio, Guangzhou, China) and one scrambled control sequence were transfected into the CT26 and MC38 cells. The efficiency of siRNA transfection was assessed by western blot analysis after 48 h of transfection. The siRNA sequences were listed as follows: CXCR3 siRNA1: 5’-GAGCAAATGTGGATGTTGT-3’; CXCR3 siRNA2: 5’-CTGCTATGCCCATATCCTA-3’; CXCR3 siRNA3: 5’-CTACGATCAGCGCCTCAAT-3’; Act1 siRNA1: Act1 siRNA2: CAGAATACTCTCCAGAAGA; CGATAGACACTGGCTATGA; Act1 siRNA3: GAACTCTAAGAACCAGCAA. The effect of siRNAs on the expression of Act1 or CXCR3 in MC38 cells was verified by western blot assay in Additional file 2: Figure S2 (A-B). Besides, the effect of CXCR3 or Act1 knockdown in MC38 on the expression of EMT markers or migration of MC38 cells was examined in different conditions (Additional file 2: Figure S2 (A-B)). siRNA1 for Act1 and siRNA2 for CXCR3 were chosen in this experiment.

### Isolation and cultures of bone marrow-derived macrophages (BMDMs) and siRNA transfection

All the following procedures were approved by the Ethics Committee of Guangdong Pharmaceutical University. Briefly, bone marrow cells were flushed from the femurs of 8 to 10-week-old anti-Act1 or wild type mice. Following the lysis of red blood cells with Ammonium-Chloride-Potassium lysis buffer (Lonza), cells were cultured with 50 ng/ml M-CSF (Pepro Tech) for 6 days in Iscove's Modified Dulbecco's Media (Gibco) supplemented with 10% heat-inactivated fetal bovine serum and 1% penicillin/streptomycin at 37 °C in a 5% CO_2_ incubator. The medium was refreshed every 3 days. For siRNA transfection, we used Lipofectamine RNAiMAX (#13778150, Thermo Fisher, MA, USA) in 6-well tissue culture plates. Briefly, on the 6^th^ day, 100 nM of three sequences of STAT3 siRNA and one scrambled control sequence were transfected into the BMDMs according to the manufacturer’s instructions. The transfected cells were cultured at 37 °C for 48 h, and then the efficiency of siRNA transfection was assessed by western blot analysis. The siRNA sequences are listed as follows: STAT3 siRNA1: 5’-CCACGTTGGTGTTTCATAA-3’; STAT3 siRNA2: 5’-GCAGGATCTAGAACAGAAA-3’; STAT3 siRNA3: 5’-GCATCAATCCTGTGGTATA-3’. The effect of siRNAs on the expression of STAT3 in anti-Act1 BMDMs was verified by western blot assay in Additional file 2: Figure S2C (a). Besides, the effect of STAT3 silencing in anti-Act1 BMDMs on the migration of MC38 cells was examined (Additional file 2: Figure S2C (b)). siRNA2 was chosen in this experiment.

For TAMs isolation and sorting from tumors of CRC patients, the detailed description in Additional file 3: Supplementary methods.

### BMDMs and CRC cell lines coculture

The coculture systems were established through 6-well plates and 0.4 μm pore size transwell inserts (Corning) [24]. Anti-Act1 or wildtype BMDMs (1 × 10^6^ cells/well) were seeded in 6-well plates and two CRC cell lines, CT26 or MC38 cells (5 × 10^5^/insert), were seeded in transwell inserts. Another coculture system exchanged the position of CT26 or MC38 cells with macrophages. For convenience, the aforementioned cells located in lower plates were regarded as research objects in the following experiments. For CD8^+^ T cells isolation, coculture with BMDMs and CRC cell lines, and flow cytometry analysis, the detailed methods were present in Additional file 3: Supplementary methods.

### In vitro wound-healing assay

MC38 cancer cells (2 × 10^5^ cells/well) were seeded in a 24-well plate and upon reaching 90 ~ 100% confluency, a linear wound was scratched with a 10 µl pipette tip. Thereafter, the transwell inserts with BMDMs were moved to the wells containing MC38 cancer cells. MC38 cell migration into the artificial wound area was monitored under microscopes. At 0 and 24 h after the creation of wounds, cell migration images were captured with a 10 × objective in a phase-contrast microscope. Cell migration was determined by the rate of cells moving toward the scratched area. ImageJ™ software was used to quantify the scratched area. All experiments were performed at least three times in triplicate.

### Transwell cell migration assay

Cell migration was further analyzed using a 24-well culture plate with polycarbonate sterile chambers (8 µm filters; Costar) without Matrigel coating. MC38 and CT26 cancer cells (2 × 10^5^ cells/insert) were incubated with 200 µl serum-free RPMI 1640 in the upper chamber and BMDMs from anti-Act1 and wild type mice in the lower chamber. The cancer cells were allowed to migrate through the upper chamber for 24 h at 37 °C. The nonmigrating cells were removed from the upper chamber with a cotton tip. Cells on the bottom side of the membrane were fixed in 4% paraformaldehyde for 30 min and stained with 0.5% crystal violet solution stain for 15 min at room temperature. Migration was quantified by counting cells in three fields of view at 40 × magnification using an inverted light microscope (Olympus). Five random fields of migrated cells were selected and counted using light microscopy.

### RNA extraction and real-time quantitative PCR (RT-qPCR) assay

Total RNA was isolated from tissue or cell samples using TRIzol Reagent (Invitrogen). cDNA was then synthesized via reverse transcription of 1 µg of total RNA using the PrimeScript RT Reagent Kit (Takara Biotechnology, Japan) according to the manufacturer’s protocols. RT-qPCR was performed with a Roche Light Cycler 2.0 (Roche Diagnostics, Basel, Switzerland). GAPDH was used as a reference housekeeping gene. All samples were tested in triplicate and repeated three times each. The 2^–ΔΔ^Ct method was used to analyze the relative mRNA expression levels. The primer sequences used for RT-qPCR are listed in Additional file 1: Table S2.

### Western blot analysis

The total cell lysate was prepared by sonicating cell extract lysed in RIPA buffer containing 50 mM Tris–HCl pH 8, 150 mM NaCl, 1% NP-40, 0.5% sodium deoxycholate, 0.1% SDS, and protease inhibitor cocktail. The protein content of the total cell extracts was determined using a Pierce BCA protein assay (Thermo Fisher Scientific). An equal amount of protein was loaded into each well of a 10% SDS-acrylamide gel, separated by gel electrophoresis, and then transferred to PVDF membranes. At room temperature, 5% skim milk blocking buffer was used to block the nitrocellulose membrane for 1 h. Then, the membrane was incubated with the primary antibody overnight at 4 °C. The next day, after washing three times with PBS, the membrane was incubated with a peroxidase-linked secondary antibody for 1 h at room temperature. Subsequently, the bands were visualized by chemiluminescence (Millipore). ImageJ (NIH, USA) was used to analyze the intensity of each band. β-actin and GAPDH were used as the internal control. The primary antibodies used were as follows: rabbit anti-E-cadherin (1:1000, #3195, Danvers, CST, MA, USA); rabbit anti-N-cadherin (1:1000, #13116, CST, Danvers, MA, USA); rabbit anti-Snail (C15D3) (1:1000, #3879, CST, Danvers, MA, USA); mouse anti-Act1(WW-18)(1:1000, #sc-100647, Santa Cruz Biotechnology, Dallas, TX, USA); rabbit anti-CXCR3 (1:100, #abs136633, Absin, Shanghai, China); rabbit anti-STAT3 (1:1000, #9139, CST, Danvers, MA, USA); rabbit anti-phospho-STAT3 (1:1000, #9145, CST, Danvers, MA, USA); rabbit anti-phospho-NF-κB p65 (Ser536) (1:1000, #3033, CST, Danvers, MA, USA); rabbit anti-NF-κB p65 (1:1000, #8242, CST, Danvers, MA, USA); goat anti-PD-L1 (1:1000, #AF1019-SP, R&D systems, USA); goat anti-CXCL10 (1:1000, #AF-466-NA, R&D systems, USA); mouse anti β-actin (1:10000, Boster, Wuhan, China) and rabbit anti-GAPDH (1:10000,CST, #2118, Danvers, MA, USA). All original blots were present in Additional file 5: Images of the original blot.

### RNA sequencing.

Total RNA in macrophages after coculture with MC38 cells was extracted using a TRIzol kit. After quantification and qualification of RNA, 1 µg RNA per sample was used as input material for the RNA sample preparations. Sequencing libraries were generated using the NEBNext® Ultra™ RNA Library Prep Kit for Illumina® (NEB, USA), and index codes were added to attribute sequences to each sample. Clustering of the index-coded samples was performed on a cBot Cluster Generation System using TruSeq PE Cluster Kit v3-cBot-HS (Illumina). After cluster generation, the library preparations were sequenced on an Illumina NovaSeq platform, and 150 bp paired-end reads were generated. Raw data (raw reads) in fastq format were first processed through in-house Perl scripts. In this step, clean data (clean reads) were obtained by removing reads containing adapters, reads containing poly-N and low-quality reads from raw data. At the same time, the Q20, Q30, and GC contents of the clean data were calculated. All downstream analyses were based on clean data with high quality. Reference genome and gene model annotation files were downloaded from the genome website directly. The index of the reference genome was built using Bowtie v2.2.3, and paired-end clean reads were aligned to the reference genome using TopHat v2.0.12. We selected TopHat as the mapping tool because TopHat can generate a database of splice junctions based on the gene model annotation file and thus gives a better mapping result than other nonsplice mapping tools. Primary data is available at (https://www.ncbi.nlm.nih.gov/geo/query/acc.cgi?acc=GSE183971).

### Bioinformatics analysis

Differential expression analysis of two groups of cocultured macrophages with MC38 cells (two biological replicates per condition) was performed using the DESeq2 R package (1.30.1). DESeq2 provides statistical routines for determining differential expression in digital gene expression data using a model based on the negative binomial distribution. The resulting P values were adjusted using Benjamini and Hochberg’s approach for controlling the false discovery rate. Genes with an adjusted P-value < 0.05 found by DESeq2 were assigned as differentially expressed. Prior to differential gene expression analysis, for each sequenced library, the read counts were adjusted by the edgeR program package through one scaling normalized factor. Differential expression analysis of two conditions was performed using the DEGSeq2 R package (1.30.1). Differential signaling pathways were enriched in cocultured macrophages from APC^Min/+^ and AA mice by GSEA software (4.0.1) and visualized using a bar plot in R. The core genes in enriched pathways were visualized by the enrichplot R package (1.12.2). Quantitative expression of differentially expressed genes in the two types of macrophages was visualized by the ComplexHeatmap R package (2.8.0).

### TCGA Data analysis

Using the R package TCGAbiolinks [25], we retrieved transcriptome profiling and clinical data of CRC patients from the TCGA database (https://portal.gdc.cancer.gov/) via project “TCGA-COAD” (Cancer Genome Atlas Colon Adenocarcinoma) and “TCGA-READ” (Cancer Genome Atlas Rectal Adenocarcinoma) in level HTSeq-counts format (Additional file 4: RDA files) and TCGAbiolinks-clinical CSV files (Additional file 4: CSV files). We used DESeq2 package to normalize HTSeq-counts data and then converted the data into Log2 normalization format (Additional file 4: XLSX files). The total retrieved data included 624 CRC patients and 51 normal samples. to show differential expression of the *ACT1* gene in CRC samples. Using the integrated repository portal for tumor-immune system interaction (TISIDB[26], http://cis.hku.hk/TISIDB/), we further retrieved the raw data of correlation between the expression of ACT1 and macrophage infiltration in CRC patients (Additional file 4: TXT files) and then calculated the correlation coefficient. As previously suggested[27], we considered the following correlation coefficients: 0.00–0.19 as very weak, 0.20–0.39 as weak, 0.40–0.59 as moderate, 0.60–0.79 as strong, and 0.80–1.0 as very strong.

### Statistical analyses

All data are presented as the mean ± standard error (SE) of independent experiments. Two-tailed one-way analysis of variance (ANOVA) with multiple comparison post hoc analysis was used, and *P* values < 0.05 (*), *P* < 0.01 (**), *P* < 0.001 (***), and *P* < 0.0001 (****) are indicated as significant. Survival analysis was performed using a log-rank test. The correlation coefficient was calculated using Spearman’s analysis. Statistical analysis was performed using GraphPad Prism 8.0 (La Jolla, CA, USA). All analyses of TCGA data were performed with R version 4.2.1 (http://www.r-project.org).

## Results

### Downregulation of ACT1 negatively correlated with TAMs numbers in CRC patients

**Fig. 1:**
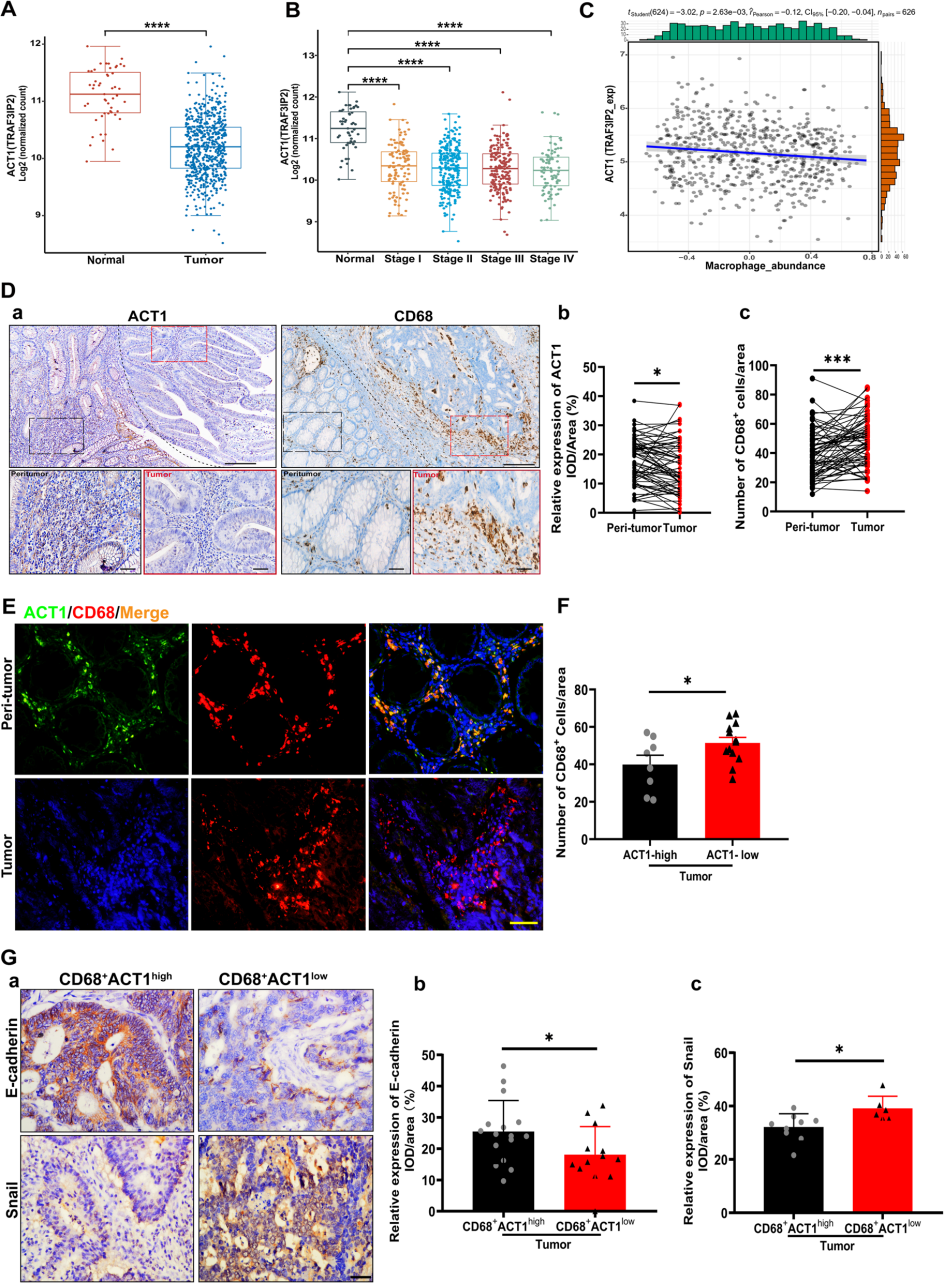
ACT1 expression in human CRC tissue and correlation with TAMs. **(A)** ACT1 expression pattern in healthy intestinal tissue (*n* = 51) and tumor tissue (*n* = 623) of colorectal adenocarcinoma patients analyzed from TCGA data. **(B)** ACT1 expression pattern in tumor tissue of distinct stages of colorectal adenocarcinoma patients analyzed from TCGA data (Normal: *n* = 51; Stage I: *n* = 111; Stage II: *n* = 238; Stage III: *n* = 184; Stage IV: *n* = 90). **(C)** The correlation of macrophage infiltration with ACT1 expression in colorectal adenocarcinoma patients analyzed from TISIDB data, *n* = 626. **(D)** Representative immunohistochemistry staining of ACT1 and CD68^+^ macrophage in the paired peri-tumor or tumor tissues of colorectal adenocarcinoma patients (*n* = 65). Peri-tumor tissues (black rectangle) and tumors (red rectangle) **(a)**. Statistical assay of ACT1 **(b)** and CD68^+^ macrophages **(c)**, respectively. Scale bar in **(a),** 200 μm (upper) and 50 μm (lower). **(E)** ACT1 colocalization with CD68^+^ macrophages in the peritumor and tumor tissues of CRC patients. Scale bar, 50 μm. **(F)** Number of CD68^+^ cells in ACT1-low expression tumor or ACT1-high tumor of CRC patients. **(G)** Immunohistochemical staining of E-cadherin and snail. Scale bar, 50 μm **(a)**. Statistical analysis of relative expression of E-cadherin **(b)** and snail **(c)** in tumor enriched CD68^+^ACT1^high^ macrophages or CD68^+^ACT1.^low^ macrophages of CRC patients. Significant difference between the groups**,** * p < 0.05, ** p < 0.01, *** p < 0.001, and **** p < 0.0001 (Student *t* test)

To investigate the role of ACT1 in CRC, we analyzed the expression pattern of ACT1 in the TCGA dataset. The expression of ACT1 was significantly downregulated in CRC patients compared with non-CRC patients (Fig. 1A). But the ACT1 level was not altered in distinct stages of CRC (Fig. 1B), strongly suggesting the essential role of ACT1 in the initial stage of CRC tumorigenesis. Furthermore, analysis from the TISIDB dataset showed that ACT1 level in tumor tissues negatively correlates with the number of CD68^+^ macrophages infiltration in CRC tumor tissues (Fig. 1C). Considering the expression of ACT1 in both intestinal epithelium and immune cells, we next detected the distribution of ACT1 and CD68^+^ macrophages in the tumor tissues of CRC patients. Notably, immunohistochemical staining showed that ACT1 is mainly expressed in the stroma and epithelium of peri-tumor tissues in CRC patients while CD68^+^ macrophages are distributed in the stroma of tumor tissues (Fig. 1D (a)). The statistical assay also demonstrated that ACT1 expressions in peri-tumor tissues are significantly higher than that in the paired tumor tissues of CRC patients (Fig. 1D (b)). On the contrary, the CD68^+^ macrophages markedly accumulated in the stroma of tumor tissues compared with peri-tumor tissues of CRC patients (Fig. 1D (c)), indicating that ACT1 downregulation in the tumor tissues might be the key factor to promote tumorigenesis and probably ACT1 knockdown contributed to the infiltration of CD68^+^ macrophages in the tumors. Besides, immunofluorescent staining results confirmed that ACT1^+^ CD68^+^ macrophages mainly localized in the stroma of the peri-tumor while the ACT1 downregulated CD68^+^ macrophages accumulated in the stroma of the tumor (Fig. 1E), suggesting that ACT1 downregulation in CD68^+^ macrophages probably contribute to the adenocarcinoma transition. Besides, the number of CD68^+^ macrophages in the stroma of the tumor was much higher in ACT1-low expression tumors than that in ACT1-high expression tumors of CRC patients (Fig. 1F). These results further indicated that ACT1 knockdown in tumor macrophages plays a key role in CRC. Furthermore, we found that relative expression of E-cadherin in the epithelium of CRC was markedly lower in the tumor enriched CD68^+^ACT1^low^ macrophages than that in the tumor-enriched CD68^+^ACT1^high^ macrophages (Fig. 1G (a-b)) and conversely, the relative expression of snail was significantly higher in the tumor enriched CD68^+^ACT1^low^ macrophages than that in the tumor enriched CD68^+^ACT1^high^ macrophages (Fig. 1G (a) and (c)). These results confirmed that ACT1 knockdown in macrophages is involved in the adenoma-adenocarcinoma transition.

### Act1 downregulation in macrophages promoted adenoma-adenocarcinoma transition and TAMs recruitment

**Fig. 2:**
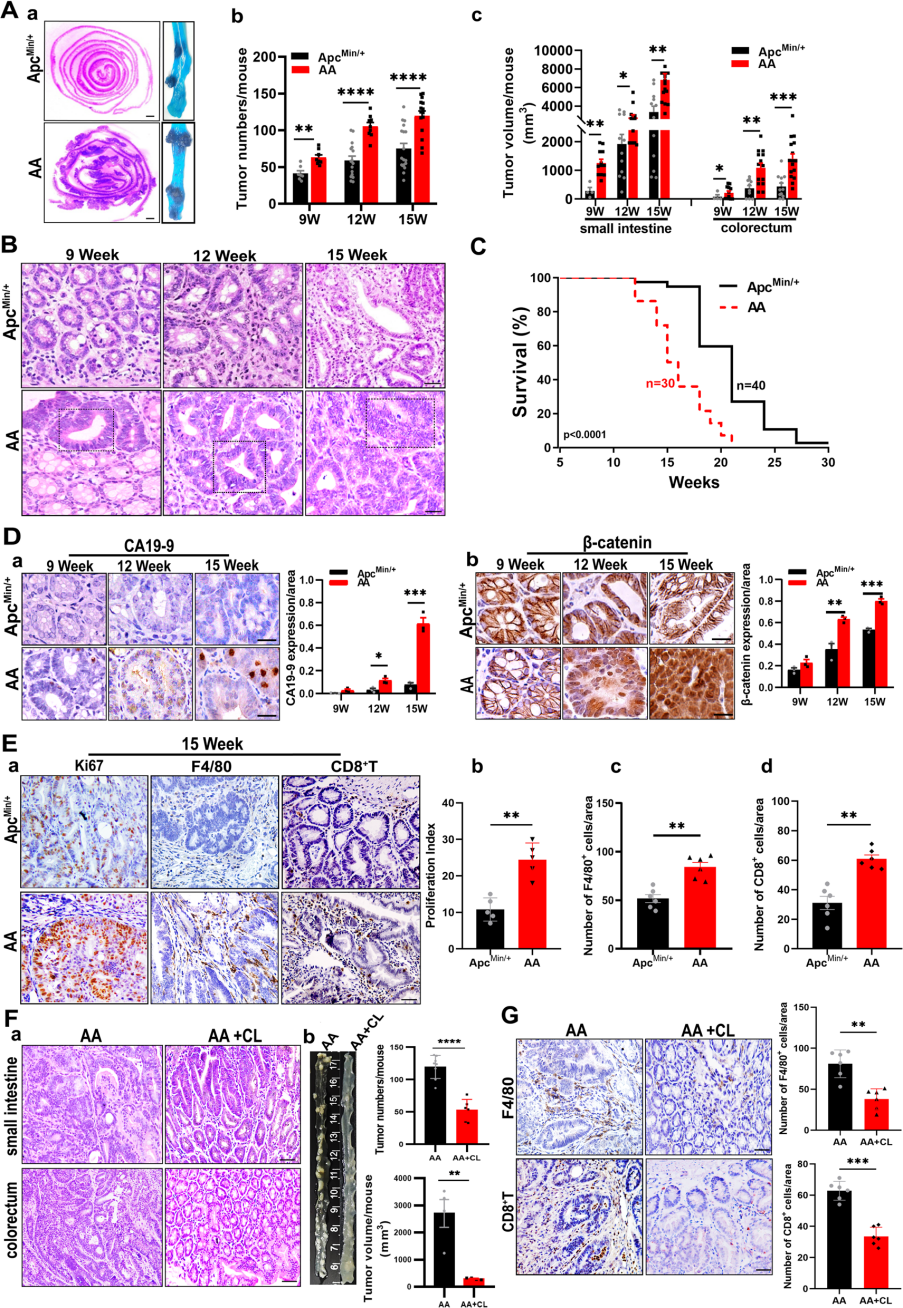
Macrophage-specific Act1 knockdown resulted in adenoma-adenocarcinoma transition in Apc^Min/+^ mice by promoting TAMs recruitment.** (A)** The effect of macrophage-specific Act1 knockdown on tumor development. **(a)** Methylene blue staining of large and many polyps of the small intestine in AA mice compared with Apc^Min/+^ mice at 15^th^ week**.** Tumor number **(b)** and tumor size **(c)** of the small intestine and colorectum in Apc^Min/+^ mice and AA mice at the 9^th^, 12^th^, and 15^th^ week. Scale bar, 1 mm. **(B)** Representative H&E staining of intestinal tumors of Apc^Min/+^ mice and AA mice at the 9^th^, 12^th^, and 15^th^ week. The dotted line box highlights adenocarcinoma transition in AA mice at different week points. Scale bar, 100 μm. **(C)** Survival analysis of Apc^Min/+^ (n = 40) and AA (n = 30) mice. The log-rank test showed a statistically significant increase in the hazard of death in AA compared with Apc^Min/+^ mice (*p* < 0.0001). **(D)** Immunohistochemical staining and statistical analysis for CA19-9 **(a)** and nuclear translocation of β-catenin **(b)** in intestinal tumors from Apc^Min/+^ and AA mice at 9^th^, 12^th^ and15^th^ week. Scale bar, 100 μm.** (E)** Representative immunohistochemistry images of Ki67, F4/80^+^ macrophages, and CD8^+^ T cells in the colon of Apc^Min/+^ mice and AA mice at the 15^th^ week. **(a)**. Scale bar, 50 μm. Quantification of Ki67 **(b)**, F4/80^+^ macrophages **(c)**, and CD8^+^ T cells **(d)**. **(F)** The effect of macrophage depletion (CL: clodronate liposomes) on tumor development in AA mice. **(a)** Representative H&E staining of tumors in Apc^Min/+^ and AA mice at the 15^th^ week. Scale bar, 100 μm. **(b)** Representative gross specimens from the indicated genotype analyzed at the 15^th^ week and quantification of tumor number and volume. **(G)** Immunohistochemical staining and quantification for F4/80^+^ macrophages and CD8^+^ T cells in tumors after macrophage depletion, Scale bar, 50 μm. Significant difference between the groups, *p < 0.05, ** p < 0.01, *** p < 0.001, and **** p < 0.0001 (Student *t* test). CL: clodronate liposomes

To further distinguish whether ACT1 in the tumor-associated macrophages is involved in the tumorigenesis, we hybridized macrophage-specific Act1 knockdown mice (anti-Act1) with spontaneous adenoma mice (Apc^Min/+^) and obtained AA mice (Additional file 2: Figure S1A). Besides, Act1 expression was remarkably reduced in primary bone-marrow-derived macrophages (BMDMs) of anti-Act1 mice (Additional file 2: Figure S1B). Subsequently, we compared the pathological change and rate of overall survival in AA mice and Apc^Min/+^ mice. The number of scattered tumors along the intestinal wall in AA mice and the total amounts of intestinal tumors at the 9^th^, 12^th^, and 15^th^ week in AA mice were significantly increased compared with those in Apc^Min/+^ mice (Fig. 2A (a-b)). Similarly, the tumor volume in distinct segments of the intestine was markedly enhanced in AA mice at different time points compared with those in Apc^Min/+^ mice (Fig. 2A (c)). Additionally, the size and weight of subcutaneous xenografts in anti-Act1 mice were much larger than those in wild type mice (Additional file 2: Figure S3 (A-B)). Meanwhile, lung metastasis nodules significantly increased in ant-Act1 mice (Additional file 2: Figure S3D (a-d)). Furthermore, the number of F4/80^+^ macrophages markedly increased in anti-Act1 mice compared with WT mice in both types of models (Additional file 2: Figure S3 (C) and D (f)). Adenocarcinoma formation which is characterized by columnar epithelial cells with elongated and nuclear pleomorphism in colonic epithelial cells only occurred at the 12^th^ week in AA mice compared to Apc^Min/+^ mice (Fig. 2B), and the adenocarcinoma alterations at the 15^th^ week worsened and were more obvious in AA mice than those in Apc^Min/+^ mice (Fig. 2B). Moreover, the rate of overall survival strikingly decreased in AA mice compared with Apc^Min/+^ mice (Fig. 2C). Meanwhile, the expression of the CRC marker, cancer antigen 199 (CA19-9) (Fig. 2D (a)), and β-catenin nuclear translocation (Fig. 2D (b)) were significantly enhanced in AA mice compared with those in Apc^Min/+^ mice at the 12^th^ and 15^th^ week. Besides, the expression of Ki67, a marker of cell proliferation, in tumor tissues was higher in AA mice than that in Apc^Min/+^ mice at the 15^th^ week (Fig. 2E (a-b)). These data verified that Act1-knockdown in macrophages promoted adenoma transition and CRC development. Furthermore, immunohistochemistry data demonstrated a prominently increased number of F4/80^+^ TAMs (Fig. 2E (c)), which is in line with the results from immunohistology staining of tumor tissues in CRC patients in Fig. 1. Besides, CD8^+^ T cells (Fig. 2E (d)) in the stroma of tumors markedly increased in AA mice compared with those in Apc^Min/+^ mice at the 15^th^ week. In contrast, macrophage depletion using clodronate liposomes (CL) completely reversed the adenocarcinoma transition (Fig. 2F (a)) and markedly reduced the size and number of tumors in AA mice (Fig. 2F (b)). Correspondingly, the number of F4/80^+^ TAMs and CD8^+^ T cells in the stroma of tumor tissues was significantly decreased in AA mice after macrophage depletion (Fig. 2G). Macrophage depletion or anti-CD8a also markedly mitigated lung metastasis in anti-Act1 mice (Additional file 2: Figure S4A (a-d)) and the number of F4/80^+^ TAMs and CD8^+^ T cells in the stroma of metastatic tissues of anti-Act1 mice (Additional file 2: Figure S4B (a-c)). Taken together, these data demonstrated that macrophage-specific Act1 knockdown indeed mediates the adenocarcinoma transition and progression of CRC via regulating TAMs function and CD8^+^ T cell infiltration.

### Act1 downregulation in macrophages facilitated epithelial-mesenchymal transition and CRC cells’ migration

**Fig. 3:**
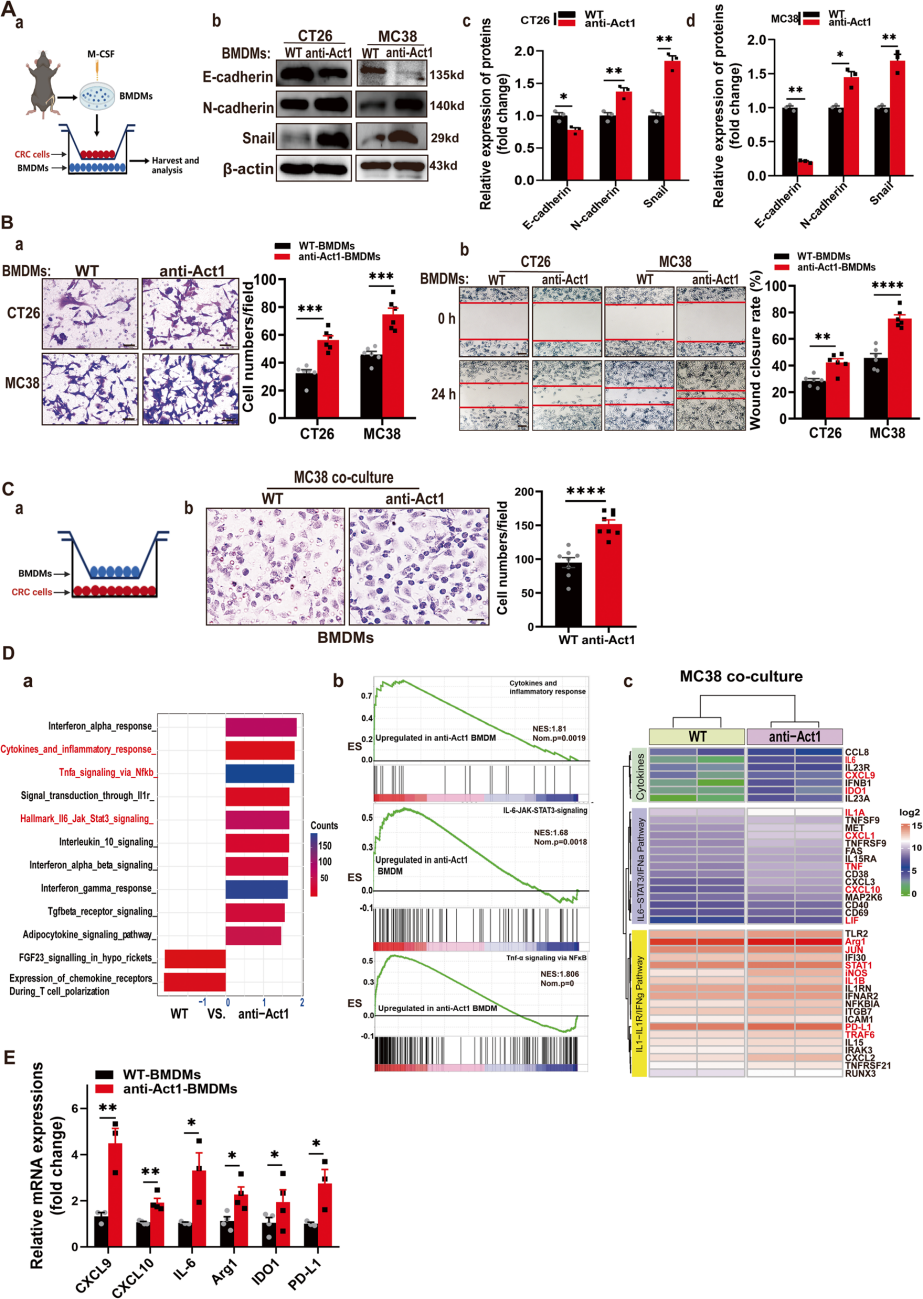
Macrophage-specific Act1 knockdown facilitated epithelial-mesenchymal transition and migration of CRC cells.** (A)** The effect of macrophage-specific Act1 knockdown on epithelial-mesenchymal transition markers in cocultured CT26 and MC38 cells. **(a)** Schematic representation of the experimental design. **(b)** Expression levels of epithelial-mesenchymal transition markers in CT26 and MC38 cells cocultured with macrophages for 48 h were evaluated by western blotting. **(c-d)** Quantification of immunoblotting results in CT26 and MC38 cells, respectively. **(B)** The effect of macrophage-specific Act1 knockdown on the migration of cocultured CRC cells. **(a)** Transwell migration assays of CT26 and MC38 cells after coculture with macrophages for 24 h. Scale bar, 50 μm. **(b)** Wound-healing assay was performed by creating a wound on a confluent monolayer of CT26 and MC38 cells during incubation with macrophages of wild type and anti-Act1 mice. Scale bar, 200 μm. **(C)** The effect of Act1 knockdown in BMDMs on the migration of BMDMs after coculture with MC38 cells for 48 h. **(a)** Schematic graph of coculture strategy; (**b)** Transwell migration assays of BMDMs after coculture with CRC cells for 24 h. Scale bar, 50 μm. **(D)** RNA sequencing of macrophages from wild type and anti-Act1 mice after coculture with MC38 cells for 24 h. **(a)** Differential GSEA enrichment of signaling pathways in macrophages of wild type and anti-Act1 mice. **(b)** Signaling pathways specifically enriched in anti-Act1 macrophage after coculture with MC38 cell line. **(c)** Differential expression of core genes in macrophages of wild type and anti-Act1 mice. **(E)** Quantitative assessment of core genes including CXCL9, CXCL10, IL-6, Arg1, IDO1,  and PD-L1 by RT-qPCR, *n* = 3. Significant difference between the groups, *p < 0.05, **p < 0.01, ***p < 0.001, and ****p < 0.0001 (Student *t* test)

To clarify whether Act1 knockdown in CRC cells promotes epithelium-mesenchyme transition, we transfected Act1 siRNA in CT26 and MC38 cells and detected EMT markers. Immunoblotting results showed that Act1 knockdown in CRC cells has no significant impact on EMT (Additional file 2: Figure S5 (A-C)), confirming that Act1 in epithelium might not be involved in the tumorigenesis of CRC. Next, to clarify the effect of Act1 in TAMs on adenoma transition via crosstalk with CRC cells, we used BMDMs from anti-Act1and wildtype mice and established a coculture system with CRC cell lines (CT26 and MC38) (Fig. 3A (a)). Western blot analysis demonstrated that anti-Act1 macrophages significantly induced the expression of epithelial-mesenchymal transition (EMT) markers such as E-cadherin, N-cadherin, and snail in CRC cells compared with those cocultured with wildtype macrophages (Fig. 3A (b-d)). Correspondingly, anti-Act1 BMDMs remarkably induced CRC cells’ migration (Fig. 3B (a-b)). Importantly, anti-Act1 BMDMs migration also significantly increased after coculture with CRC cells compared with wildtype BMDMs (Fig. 3C (a-b)). Whereas Act1-knockdown in CRC cell lines did not significantly affect wildtype BMDMs recruitment (Additional file 2: Figure S5D (a-b)). These results further confirmed that Act1 knockdown in macrophages but not in CRC cells plays a key role in the regulation of the interaction with the CRC cells and the underlying mechanism was probably cytokines-mediated paracrine effects. Moreover, GSEA results from RNA sequencing showed that differentially expressed core genes in the anti-Act1 macrophages cocultured with CRC cells were enriched in the IFNα response, IL6-STAT3, TNFα-NF-κB, and IL-1R signal pathways (Fig. 3D (a-c)). Notably, RT-qPCR analysis further confirmed higher expression of CXCL9/10, IL-6, Arg1, IDO1, and PD-L1 in anti-Act1 macrophages cocultured with CRC cells than wildtype macrophages cultured with CRC cells (Fig. 3E). Arg1, IDO1, and PD-L1 are also expressed in myeloid-derived suppressor cells (MDSCs) [28, 29], indicating the immunosuppressive phenotype of anti-Act1 macrophages after coculture with CRC cells. Similarly, using TAMs from CRC patients (Additional file 1: Table S3) with low or high ACT1 expression (Additional file 2: Figure S6A), we demonstrated that CXCL9, CXCL10, and PD-L1 significantly increased in ACT1-low TAMs compared with that of ACT1-high TAMs (Additional file 2: Figure S6B). Besides, ACT1-low TAMs markedly promote CRC cells migration (Additional file 2: Figure S6C (a-b)) and the mRNA expression of EMT markers compared with ACT1-high TAMs (Additional file 2: Figure S6C (c)), which were in line with our results obtained from BMDMs.

### Act1 downregulation in macrophages activates STAT3 to promote adenoma-adenocarcinoma transition via CXCL9/10-CXCR3-axis in CRC cells and immunosuppression via PD-l/PDL-1axis in CD8^+^ T cells

**Fig. 4:**
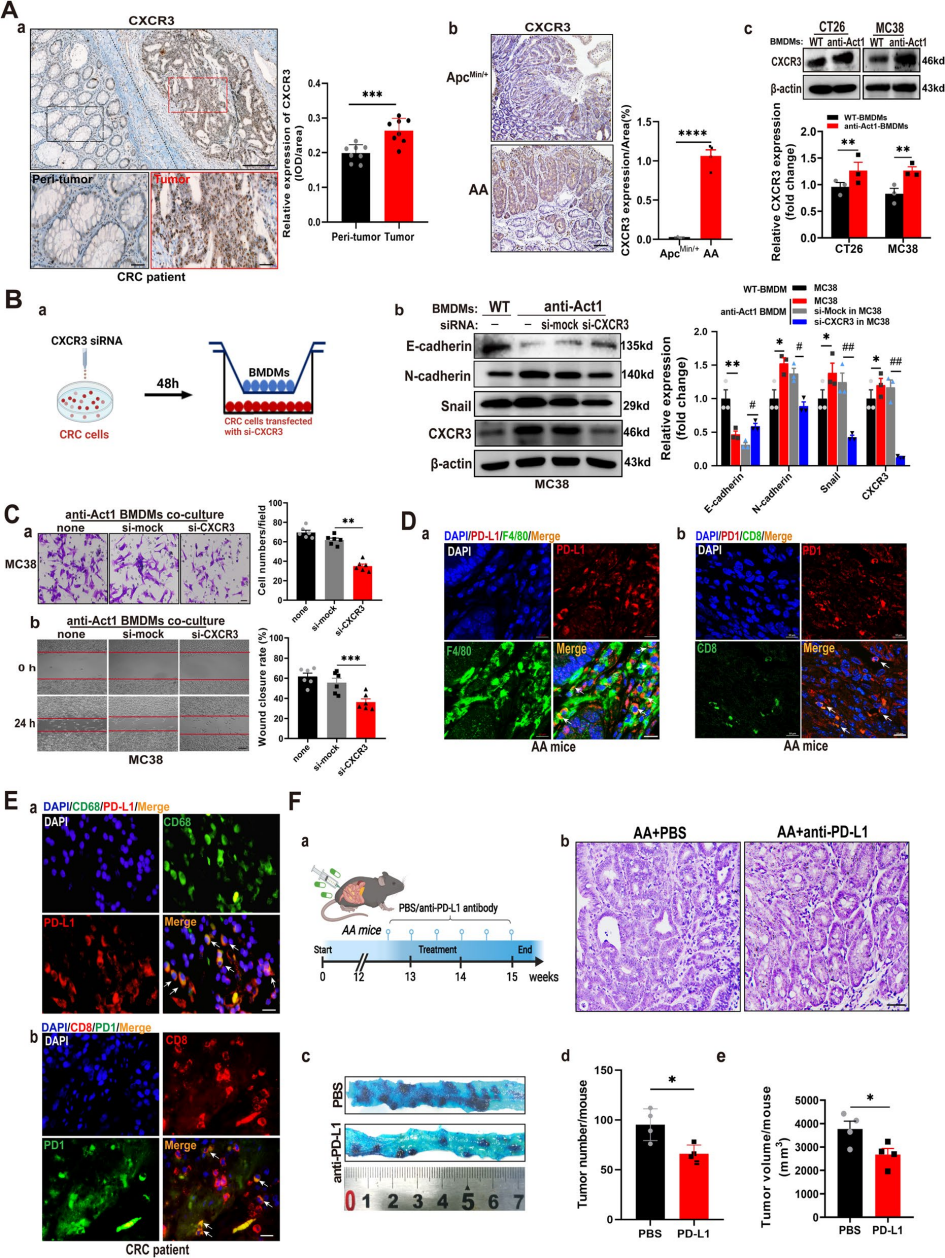
Act1-knockdown in macrophages promoted epithelial-mesenchymal transition and CRC cell migration via activating CXCL9/10/11-CXCR3 and immunosuppression via PD1/PD-L1 axis. **(A)** CXCR3 expression was assayed by immunohistochemical staining in the tumor tissue of CRC patients, *n* = 6 **(a)**, tumor of Apc^Min/+^ and AA mice at 15^th^ week **(b)**, and CT26 or MC38 cells cocultured with BMDMs of wild type and anti-Act1 mice for 48 h **(c)**. Scale bar in **(a),** 200 μm (upper) and 50 μm (lower). Scale bar in **(b)**, 100 μm. **(B)** The effect of cocultured macrophages on the migration of CXCR3-knockdown MC38 cells. Schematic representation of the experimental design **(a)**. Expression levels of epithelial-mesenchymal transition markers in CXCR3 knockdown MC38 cells cocultured with wild type or anti-Act1 macrophages for 48 h **(b)**. **(C)** The effect of CXCR3 knockdown in MC38 cells on macrophage-mediated migration of MC38 cells. (**a)** Transwell migration assay of MC38 cells after coculture with anti-Act1 macrophages for 24 h. Scale bar, 50 μm. **(b)** Wound-healing assay of MC38 cells during incubation with macrophages of anti-Act1 mice for 0 h and 24 h. Scale bar, 200 μm**. (D)** Immunofluorescence staining for PD-L1 (red) and F4/80 (green) **(a)**, PD1 (green) and CD8^+^ T (red) **(b)** in AA mice at the 15^th^ week was taken by laser confocal microscope. Scale bars, 10 μm. (**E)** Immunofluorescence staining for PD-L1 (red) and CD68^+^ (green) **(a)**, PD1 (green) and CD8^+^ T (red) **(b)** in CRC patients was taken by an immunofluorescent microscope. Scale bars, 10 μm. **(F)** The effect of anti-PD-L1 on the adenocarcinoma transition in AA mice after treatment 3 weeks. (**a)** Graphical depiction of anti-PD-L1 treatment. (**b)** Representative H&E staining of intestinal tumors of AA mice treated with PBS or anti-PD-L1. Scale bar, 100 μm. (**c)** Methylene blue staining of tumors in the small intestine from AA mice treated with PBS or anti-PD-L1 after 3 weeks**. (d-e)** Quantification of tumor number and tumor size in AA mice after 3 weeks of anti-PD-L1 treatment, *n* = 3. *p < 0.05, ^#^*p* < 0.05, **p < 0.01, ^##^*p* < 0.01, ***p < 0.001, and ****p < 0.0001 are considered statistically significant

Studies demonstrated that CXCL9/10/11 induced epithelial-mesenchymal transition of CRC cells via acting on their common receptor CXCR3 [30]. Therefore, we examined the expression of CXCR3 in CRC patients and both types of mice. Immunohistochemical staining showed that CXCR3 was highly distributed in the tumor epithelium of CRC patients compared with that of peri-tumor tissues (Fig. 4A (a)). Similarly, CXCR3 expression in adenocarcinoma tissues of AA mice was markedly enhanced compared with adenoma tissues in Apc^Min/+^ mice (Fig. 4A (b)). Furthermore, Act1-knockdown in macrophages remarkably induced CXCR3 expression in CRC cells compared with wild type macrophages (Fig. 4A (c)). Importantly, silencing CXCR3 in CRC cells effectively mitigated anti-Act1 macrophage-induced epithelial-mesenchymal transition (Fig. 4B (a-b)) and migration of CRC cells (Fig. 4C (a-b)). These results strongly indicated that Act1 downregulated macrophages crossed talk with CRC cells via the interaction of CXCL9/10 and CXCR3.

Our previous result showed that anti-CD8a inhibits lung metastasis in anti-Act1 mice, suggesting that Act1 knockdown in macrophages probably interact with CD8^+^T cell. Consequently, by adding CD8^+^ T cells in the coculture system, we found Act1-knockdown in BMDMs significantly increased the number of PD1^+^Tim3^+^CD8^+^ T cells compared with wild type-BMDMs (Additional file 2: Figure S7), strongly indicating that macrophage-specific Act1 knockdown may be exhausted CD8^+^ T cells. Moreover, immunofluorescent staining showed that PD-L1(red) indeed colocalized with F4/80^+^ macrophage (green) in the stroma of tumor tissue of AA mice (Fig. 4D (a)). Meanwhile, double-positive of PD1 (red) and CD8 (green) T cells were also located in the stroma of tumors in AA mice (Fig. 4D (b)). Similarly, colocalization of CD68^+^ macrophages (green) and PD-L1 (red) (Fig. 4E (a)) was mainly distributed in the stroma of tumor tissues in CRC patients as well as both CD8^+^ T cells (red) and PD1(green) (Fig. 4E (b)). Consequently, anti-PD-L1 (Atezolizumab) was administrated to AA mice in the schematic graph (Fig. 4F (a)). Atezolizumab suppressed adenocarcinoma transition in AA mice (Fig. 4F (b)) and consequently reduced the number (Fig. 4F (c-d)) and volume (Fig. 4F (e)) of tumors, which substantiated the immunosuppressive features of TAMs in AA mice.

**Fig. 5:**
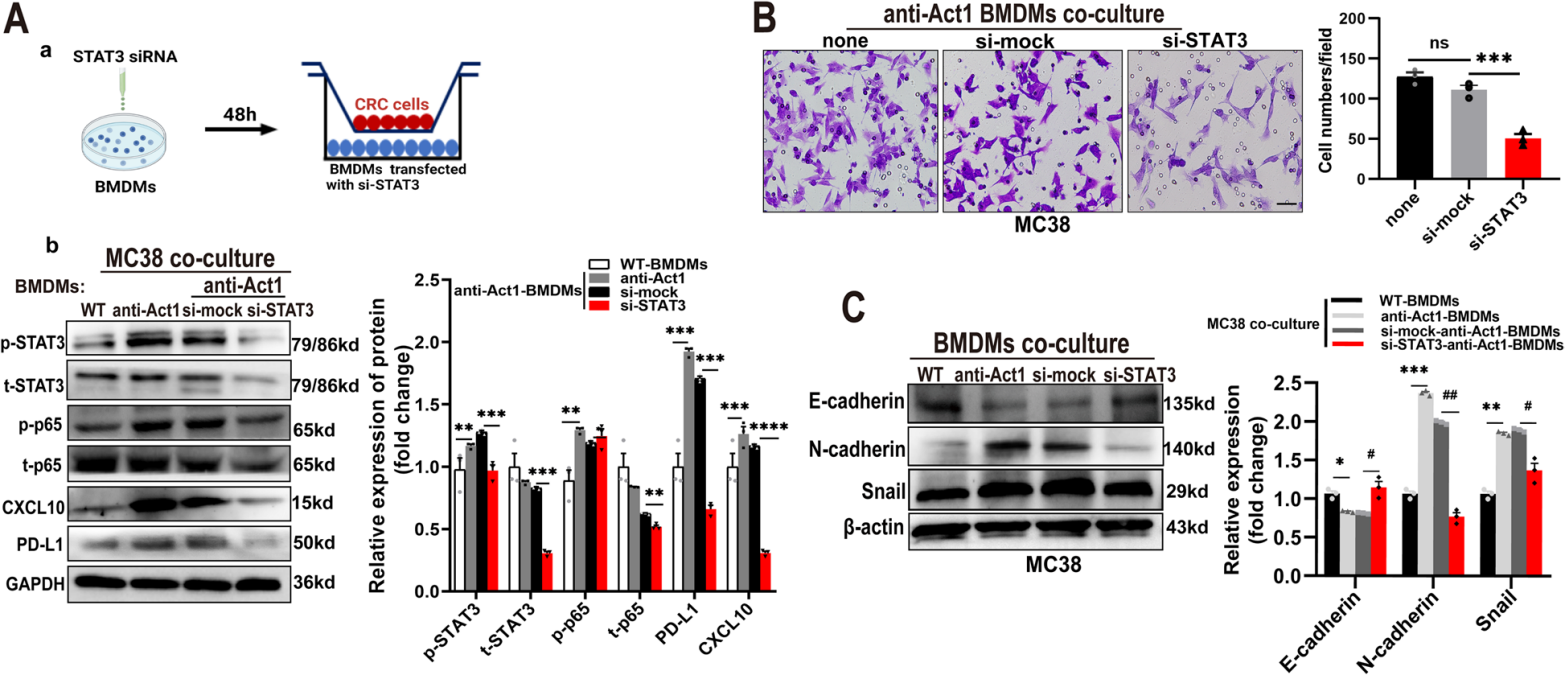
Macrophage-specific Act1-knockdown increased CXCLs secretion and PD-L1 expression via STAT3 activation. **A. (a)** Schematic representation of the experimental design.** (b)** The effect of STAT3 silencing in anti-Act1 macrophages Representative western blot images of pSTAT3, p-p65, CXCL10, and PD-L1 expression and quantification in anti-Act1 macrophages cocultured with MC38 cells. (**B**) The effect of STAT3 silencing in anti-Act1 macrophages on migration after coculture with MC38 cells for 24 h. Scale bar, 50 μm. (**C)** Representative western blot images and quantification of epithelial-mesenchymal transition markers in MC38 cells cocultured with macrophages, *n* = 3. Significant difference between the groups, *p < 0.05, ^#^*p* < 0.05, ** p < 0.01, ^##^*p* < 0.01, ***p < 0.001, and ****p < 0.0001. GAPDH or β-actin was used as an internal loading control

**Fig. 6:**
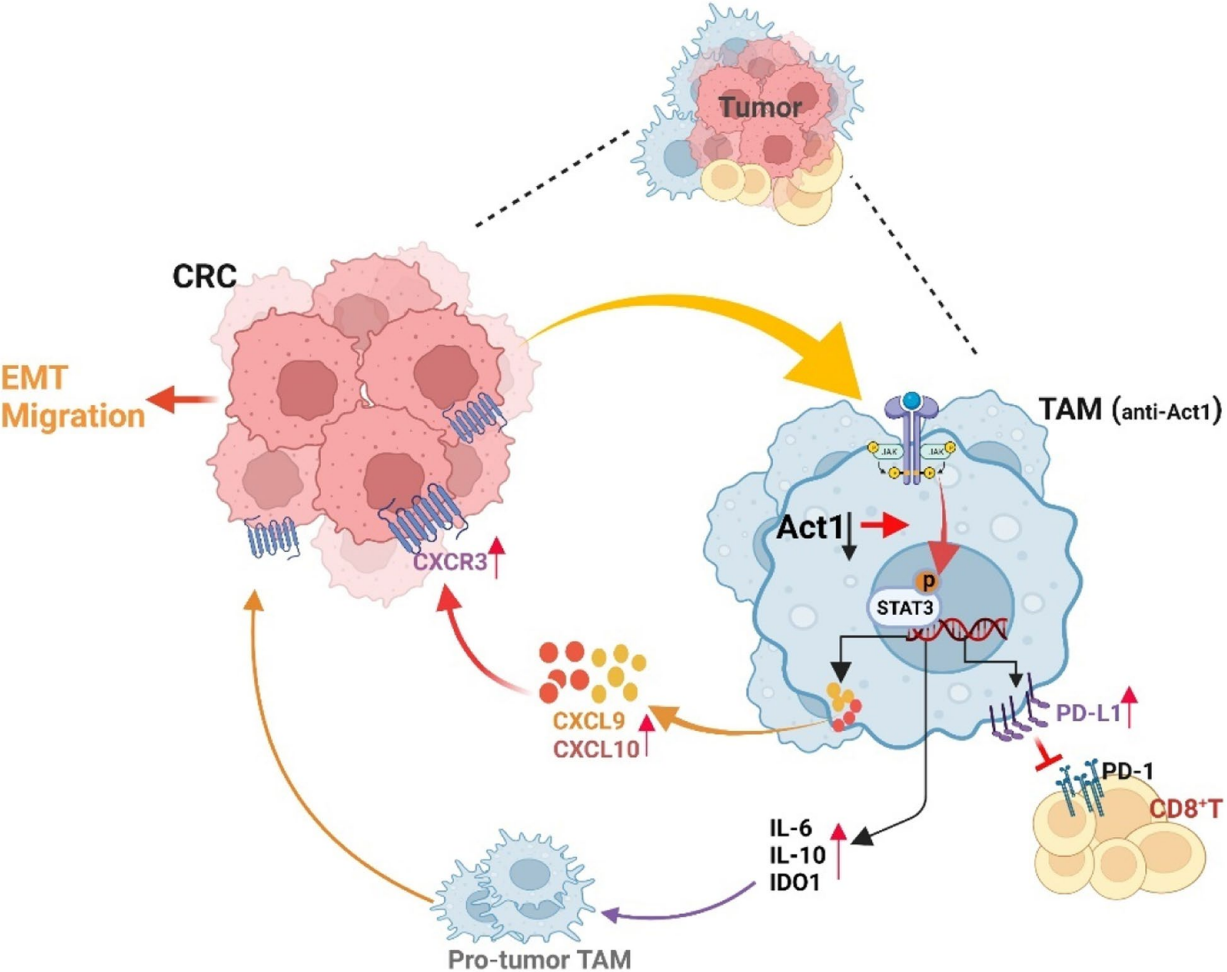
Regulation of CRC progression by macrophage-specific Act1 knockdown. Macrophage-specific Act1 knockdown promoted adenocarcinoma transition through the recruitment of TAMs and CD8^+^ T cells in tumor tissues. By activating STAT3 transcription factors, macrophages-specific Act1knockdown crosstalk with CRC cells and CD8^+^ T cells via CXCL9/10-CXCR3 and PD-1/PD-L1 axis and consequently promoted epithelial-mesenchymal transition and CRC progression. (Figure was created with Biorender.com)

A recent study indicated that STAT3 activation is involved in CXCL9/10 secretion from TAMs and is closely associated with anti-PD-L1 treatment in CRC [31]. Besides, TNFα-NF-κB and IL-6-STAT3 signaling pathways are collaboratively involved in promoting endothelial-mesenchymal transition and tumor progression [32–34]. In line with results from the literature, our data showed that CRC cells induced IL6-STAT3 and TNFα-NF-κB signaling pathways in anti-Act1 macrophages compared with wild type macrophages. Therefore, we here examine the role of CRC-activated STAT3 and NF-κB in macrophages in CRC development. The experimental design is shown in Fig. 5A (a). Notably, Western blot assays showed that coculture MC38 cells markedly increase p65 phosphorylation in anti-Act1macrophages compared with wildtype macrophages (Fig. 5A (b)), suggesting that NF-κB activation perhaps did not only rely on Act1 under coculture condition. Interestingly, CRC cells significantly induced STAT3 activation in anti-Act1 macrophages compared with that in wild type macrophages (Fig. 5A (b)). Meanwhile, STAT3 knockdown in anti-Act1 macrophages markedly reduced CRC cell-induced total p65 expression in anti-Act1 macrophages, suggesting STAT3-mediated regulation of NF-κB. Furthermore, our results revealed that silencing STAT3 in anti-Act1 macrophages markedly inhibited CRC cells-induced CXCL10 and PD-L1 expression in anti-Act1 macrophages (Fig. 5A (b)). Correspondingly, downregulation of STAT3 in anti-Act1 macrophages dramatically reduced cocultured CRC cells’ migration (Fig. 5B). Correspondingly, the expressions of EMT markers such as E-cadherin, N-cadherin, and snail were significantly reduced in CRC cells cocultured with STAT3 downregulated anti-Act1 macrophages (Fig. 5C). Taken together, our findings confirmed that Act1 downregulation in macrophages activates STAT3 to promote adenoma-adenocarcinoma transition via CXCL9/10-CXCR3-axis in CRC cells and immunosuppression via PD-l/PD-L1 axis in CD8^+^ T cells (Fig. 6).

## Discussion

TAMs are the major component of tumor-associated stromal cells that orchestrate cancer-related inflammation [35]. TAMs induce CD8^+^ T cell exhaustion via PD1/PD-L1 interaction to exacerbate the development of cancer [36]. By TCGA and TISIDB analysis, we noticed the downregulation of Act1 expression in tumor tissues of CRC patients negatively correlated with accumulated CD68^+^ macrophages in the tumor. Confirmatively, the number of ACT1^low^CD68^+^ macrophages markedly accumulated in the stroma of tumor of CRC patients. Besides, relative expression of EMT markers in the tumor enriched ACT1^low^CD68^+^ macrophages of CRC patients further suggested that ACT1 knockdown in macrophages involved in adenoma-adenocarcinoma transition. Notably, we first distinguished that macrophage-specific Act1 knockdown strikingly promoted adenoma-adenocarcinoma transition, shortened the overall survival of Apc^Min/+^ mice, and lung metastasis via the accumulation of immunosuppressive TAMs and exhaustive CD8^+^ T cells in the stroma of the tumor. Importantly, macrophage depletion effectively reversed the adenoma-adenocarcinoma transition in AA mice and inhibited lung metastasis in anti-Act1 mice. Neutralization CD8a also markedly mitigated lung metastasis in anti-Act1 mice. Moreover, the PD-L1 inhibitor mitigated the effect of Act1 knocked down macrophages on the adenoma transition. Using a coculture system, we further illustrated that Act1-knockdown significantly enhanced CXCL10 and PD-L1 expression and promoted an immunosuppressive phenotype in macrophages via preferentially activating STAT3 transcription factor. Therefore, macrophage-specific Act1 knockdown modulated adenoma transition and CRC progression by orchestrating CXCL9/10-CXCR3 and PD1/PD-L1 axis to cross-talk with CRC cells and CD8^+^ T cells in the tumor microenvironment.

Prominent immune cell infiltration and enhanced expression of inflammatory cytokines and chemokines are observed in the majority of sporadic CRC tissues [37]. With high heterogeneity and plasticity in the tumor microenvironment, TAMs play either a tumor-promoting or tumor-preventive role in response to different stimuli [38]. As a regulatory subunit of IKKs, NEMO/IKKγ is an essential modulator for NF-κB activation while Act1, a NEMO/IKKγ-associated protein, connects to both the IKK and JNK signaling complexes to mediate their activation [18]. Besides, Act1 is absolutely required for IL-17 induced TRAF6/NF-κB activation [39]. Notably, our RNA-seq results suggested that Act1 knockdown in macrophage cocultured with CRC cells specifically enriched cytokines (IL-6, CXCL9/10, CXCL3, CXCL2, IFNβ1, TNF, IL-1β, IL-1α, etc.) expression related to TNF-α-NF-κB, cytokines-cytokines receptors, and IL-6-STAT3 signaling, suggesting the role of macrophagic Act1 in inflammation regulation. Besides, inflammatory mediators such as TNF, IL-1β, IL-6, IL-11, and IL-8, are reportedly potent inducers of epithelial-mesenchymal transition [40]. Consistently, we found that Act1 knockdown in macrophages promoted epithelial-mesenchymal transition and the development of CRC in Apc^Min/+^ mice.

Studies in various inflammation-induced murine tumor models have demonstrated the requirement of macrophagic NF-κB activation for tumor promotion [41–43]. Our findings demonstrated that coculture CRC cells enhanced NF-κB activation in both types of BMDMs whereas the extent of NF-κB activation was even higher in anti-Act1 BMDMs than that in wildtype BMDMs, indicating that Act1 probably was not a sole regulator of NF-κB activation in macrophages. Furthermore, we demonstrated that Act1 knockdown in BMDMs increased NF-κB activation and the expression of PD-L1 and CXCL10 via upregulating STAT3 phosphorylation after coculture with MC38 cell line compared to those in wildtype BMDMs, strongly suggesting that Act1 knockdown contributes to the interaction between p65 and STAT3 in BMDMs. Studies demonstrated that Act1 is able to directly interact with and suppress STAT3 activation in Th17 cells and therefore Act1 knockout activates STAT3 signaling pathway [44]. STAT3 is constitutively expressed in both immune and malignant cells and is an important mediator of the immunosuppressive inflammatory response in the tumor microenvironment and the tumor immune escape process [45]. Meanwhile, with the coordination of NF-κB and STAT3, TAMs often prime their cancer-fighting phenotypes toward a cooperative tumor development profile [46]. Mechanistically, p65 physiologically interacts with STAT3 and consequently modifies their transcriptional activity [47]. Besides, unphosphorylated NF-κB interacts with STAT3 cooperatively and then binds to the κB elements of promoters such as CCL5 and IL-6 to collaboratively induce their target gene expression [48]. Importantly, we found that silencing STAT3 in anti-Act1 macrophages effectively reduced total p65 expression, confirming that Act1 knockdown strengthens the interaction between STAT3 and NF-κB. Collectively, we speculated that Act1 knockdown in BMDMs probably removes the inhibition of STAT3 and then STAT3 activation promotes physiological interaction with p65 which increases p65 activation and the expression of p65 to enhance their transcript activity.

Moreover, both STAT3 and NF-κB transcription factors also synergistically control a common set of genes encoding for cytokines and chemokines[49]. CXCL9, CXCL-10, and CXCL-11 are mainly secreted by monocytes, endothelial cells, fibroblasts, and cancer cells in response to IFN-γ, which are synergistically enhanced by TNF-alpha while CXCR3, a common receptor of CXCL9/10/11, is preferentially expressed on the surface of monocytes, T cells, NK cells, dendritic cells, and cancer cells [30]. CXCL9/10-CXCR3 interactions govern immune infiltration into tumors prior to therapy[50]. Our data indicate that Act1 knockdown significantly promoted CXCL10 expression in macrophages cocultured with CRC cells via hyperactivation of STAT3 and correspondingly mediated migration of CRC cells via CXCR3. A recent study showed that CXCL9 and CXCL10 from macrophages are critical for the recruitment of T cells in the tumor microenvironment [51]. Similarly, we also observed that macrophage depletion effectively reduced the recruitment of CD8^+^ T cells and adenoma transition in AA mice, confirming the crucial effect of cytokines from anti-Act1 macrophages on the adenocarcinoma occurrence. Besides, IL-6 and TNF-α secreted from macrophages induce PD-L1 expression through NF-κB and STAT3 signaling pathways in gastric cancer cells [52]. Furthermore, STAT3 hyperactivation in immune cells also mediates inhibition of the innate and adaptive immunity of the tumor microenviroment [53]. Our data demonstrated that Act1 knockdown promoted PD-L1 expression in macrophages via STAT3 activation in the coculture system. Importantly, Act1 knockdown in BMDMs significantly increased PD1^+^ Tim3^+^ exhausted CD8^+^ T cell after coculture with CRC cells compared with that of wildtype BMDMs. Correspondingly, anti-CD8a treatment effectively alleviated lung metastasis in anti-Act1 mice. Moreover, anti-PD-L1 markedly reduced the amounts of tumor and adenoma transition in AA mice. Therefore, our results confirmed the effect of Act1 in macrophages on the chemokines secretion, immunosuppressive phenotype, and immune checkpoint blockade in the tumor microenvironment.

## Conclusions

We characterized the population of macrophages with Act1 knockdown that fosters adenocarcinoma growth and progression. The AA mice would be the optional model for the investigation of CRC tumorigenesis. Importantly, our findings indicated that macrophage-specific Act1 knockdown activates STAT3 transcription factors to crosstalk with CRC cells and CD8^+^ T cells via CXCL9/10-CXCR3 and PD-1/PD-L1 axis respectively, and consequently promotes epithelial-mesenchymal transition and CRC progression. Intriguingly, Act1 downregulation in TAMs could be a promising predictor of favorable prognosis for anti-PD-L1 therapy in CRC patients.

## Supplementary Information

**Additional file 1:  TableS1.** Clinicopathological characteristics of colorectum cancer patients in Fig. 1.**Table S2.** Primers used for RT-qPCR in the study. **Table S3.** Demographics andclinical information of colorectal patients in Figure S7.

**Additional file 2: Figure S1.** Genotyping assays and Act1 expression in both types of mice. **Figure S2.** Theeffect of siRNAs on the expression of Act1, CXCR3, and STAT3 in correspondingcells. **Figure S3.** The effect of macrophage-specific Act1 knockdown on a xenograftmodel of CRC cells and lung metastasis mouse model of CRC. **Figure S4.** The effectof macrophage depletion or anti-CD8a therapy in the lung metastasis mouse modelof CRC two weeks after being administrated with MC38 cells. **Figure S5.** The effectof Act1 knockdown in CRC cells on the expression of EMT Markers. **Figure S6.** Theeffects of TAMs from CRC patients on the migration and EMT of CRC cell line. **Figure S7.** The effect of Act1-knockdown in BMDMs on the CD8+ T cells exhaustion.

**Additional file 3.** Supplementarymethods.

**Additional file 4.** rawdata retrieved from TCGA and TISIDB.

**Additional file 5.** Imagesof the original blot.

## Data Availability

The data discussed in this publication have been deposited in NCBI's Gene Expression Omnibus [54] and are accessible through GEO Series accession number GSE183971 (https://www.ncbi.nlm.nih.gov/geo/query/acc.cgi?acc=GSE183971).

## Abbreviations

CRC: Colorectal cancer
TAMs: Tumor-associated macrophages
NF-κB activator1: Act1
anti-Act1: Macrophage-specific Act1-knockdown
AA: Apc^Min/+^; anti-Act1
APC: Adenomatous polyposis coli
TRAF3IP2: TRAF3-interacting protein 2
PBS: Phosphate-buffered saline
BMDMs: Bone marrow-derived macrophages
CL: Clodronate liposomes
PD-L1: Programmed cell death ligand 1
PD-1: Programmed cell death protein 1
EMT: Epithelial-mesenchymal transition
MDSCs: Myeloid-derived suppressor cells
FACS: Fluorescence-activated cell sorting
