# Supplementary material for: NF-κB Activator 1 downregulation in macrophages activates STAT3 to promote adenoma-adenocarcinoma transition and immunosuppression in colorectal cancer
Source: BMC Med. 2023 Mar 29;21:115. doi: 10.1186/s12916-023-02791-0 (PMC10053426; doi:10.1186/s12916-023-02791-0)
Supplement: Supplementary file 1 — Additional file 1: TableS1. Clinicopathological characteristics of colorectum cancer patients in Fig. 1.Table S2. Primers used for RT-qPCR in the study. Table S3. Demographics andclinical information of colorectal patients in Figure S7. [file 12916_2023_2791_MOESM1_ESM.docx]

**Supplementary Tables**

**Table S1** Clinicopathological characteristics of colorectum cancer patients (n=65)

| Variables | No. and (%) |
| --- | --- |
| No. of patients | 65 |
| Age (median; range), years | 66; 33-87 |
| Gender (male/female) | 40/25 (61.5/38.5) |
| Tumor size, cm (≤5/>5) | 30/35(46.2/53.8) |
| Tumor location (colon/rectum) | 50/15 (76.9/23.1) |
| TNM stage (Ⅰ-Ⅱ/Ⅲ-Ⅳ) | 30/35(46.2/53.8) |
| Differentiation (high/middle) | 7/58 (10.8/89.2) |

**Table S2** Primers used for RT-qPCR

| **Gene** | **Forward 5-3’** | **Reverse 5-3’** |
| --- | --- | --- |
| **GAPDH (***mus musculus***)** | TGTTTGTGATGGGTGTG | TACTTGGCAGGTTTCTC |
| **CXCL9 (***mus musculus***)** | GAACGGAGATCAAACCTGCCT | TGTAGTCTTCCTTGAACGACGA |
| **CXCL10 (***mus musculus***)** | CGATGACGGGCCAGTGAGAATG | TCAACACGTGGGCAGGATAGGCT |
| **IL-6 (***mus musculus***)** | TAGTCCTTCCTACCCCAATTTCC | TTGGTCCTTAGCCACTCCTTC |
| **Arg1 (***mus musculus***)** | CATTGGCTTGCGAGACGTAG | TTGCCAATCCCCAGCTTGTC |
| **IDO1 (***mus musculus***)** | CTGACCACCTCAGGGTTTAGC | TTTCTAGCCACAAGGACCCAG |
| **PD-L1 (***mus musculus***)** | GCTTCTCAATGTGACCAGCA | GAGGAGGACCGTGGACACTA |
| **GAPDH (***homo sapiens***)** | CGGACCAATACGACCAAATCCG | AGCCACATCGCTCAGACACC |
| **E-cadherin (***homo sapiens***)** | TACGCCTGGGACTCCACCTA | CCAGAAACGGAGGCCTGAT |
| **Vimentin (***homo sapiens***)** | GCTAACCAACGACAAAGCCC | GATTGCAGGGTGTTTTCGGC |
| **Snail** *(homo sapiens)* | CACTATGCCGCGCTCTTTC | GCTGGAAGGTAAACTCTGGATTAGA |
| **CXCL9 (***homo sapiens***)** | AAGACCTTAAACAATTTGCCCC | TGCTGAATCTGGGTTTAGACAT |
| **CXCL10 (***homo sapiens***)** | CTCTCTCTAGAACTGTACGCTG | ATTCAGACATCTCTTCTCACCC |
| **PD-L1 (***homo sapiens***)** | CCTACTGGCATTTGCTGAACGCAT | ACCATAGCTGATCATGCAGCGGTA |

**Table S3** Demographics and clinical information of colorectal patients.

| **CRC patient** | **Age (yrs)** | **Gender** | **TNM stage** | **Diagnosis** |
| --- | --- | --- | --- | --- |
| **1** | 54 | male | T3N1M0 | Colon cancer |
| **2** | 58 | male | T3N0M0 | Colon cancer |
| **3** | 35 | male | T4aN2M1 | Colon cancer |
| **4** | 59 | male | T3N0M0 | Colon cancer |
| **5** | 59 | female | T3N0M0 | Colon cancer |
| **6** | 73 | male | T3N2M1 | Colon cancer |
| **7** | 64 | male | T3N0M0 | Colon cancer |
