# Supplementary material for: NF-κB Activator 1 downregulation in macrophages activates STAT3 to promote adenoma-adenocarcinoma transition and immunosuppression in colorectal cancer
Source: BMC Med. 2023 Mar 29;21:115. doi: 10.1186/s12916-023-02791-0 (PMC10053426; doi:10.1186/s12916-023-02791-0)
Supplement: Supplementary file 2 — Additional file 2: Figure S1. Genotyping assays and Act1 expression in both types of mice. Figure S2. Theeffect of siRNAs on the expression of Act1, CXCR3, and STAT3 in correspondingcells. Figure S3. The effect of macrophage-specific Act1 knockdown on a xenograftmodel of CRC cells and lung metastasis mouse model of CRC. Figure S4. The effectof macrophage depletion or anti-CD8a therapy in the lung metastasis mouse modelof CRC two weeks after being administrated with MC38 cells. Figure S5. The effectof Act1 knockdown in CRC cells on the expression of EMT Markers. Figure S6. Theeffects of TAMs from CRC patients on the migration and EMT of CRC cell line. Figure S7. The effect of Act1-knockdown in BMDMs on the CD8+ T cells exhaustion. [file 12916_2023_2791_MOESM2_ESM.docx]

**Supplemental figures and figure legends**

**
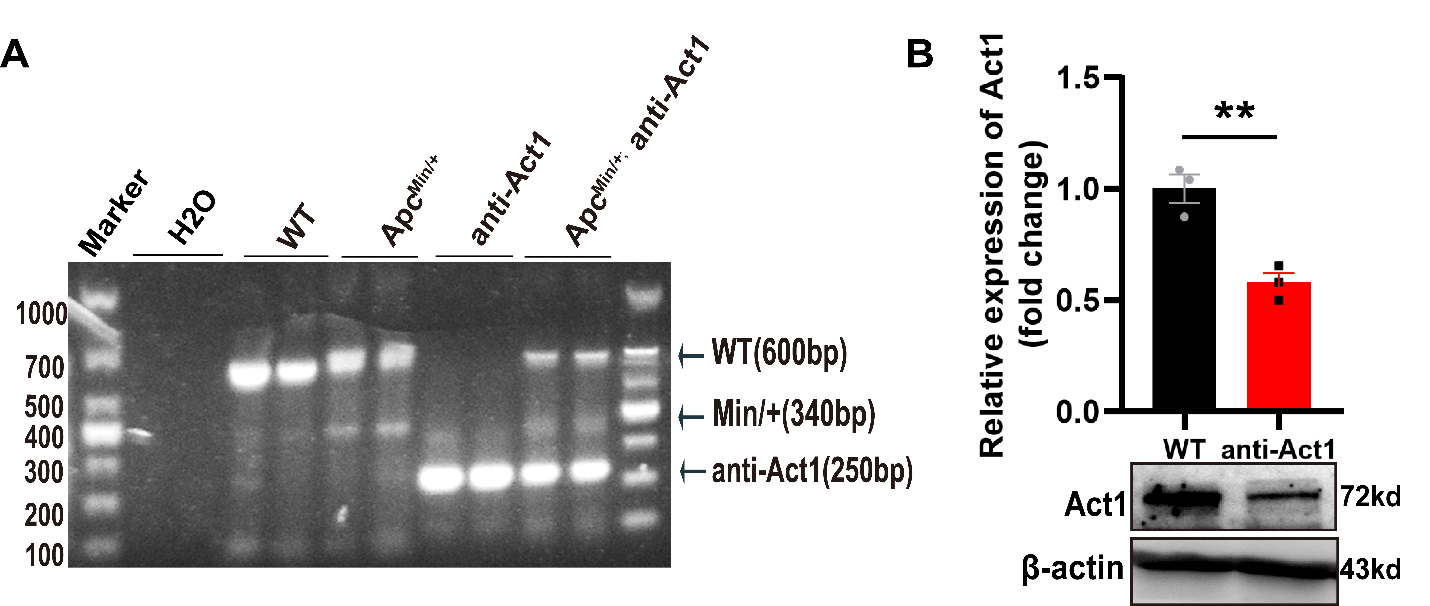
**

**Fig. S1 Genotyping assays and Act1 expression in both types of mice.**

**A.** PCR amplicons from genomic DNA obtained by tail biopsies from mice of the indicated genotype. Bands of different sizes correspond to the Apc^Min/+^ allele (340 bp), anti-Act1 allele (250bp), and wildtype allele (600bp); **B.** Act1 expression in bone marrow-derived macrophage (BMDM) from two types of mice. Data are shown as means ± s.e.m. of three independent experiments with at least three independent measurements/experiment. Significant difference between the groups, ***p*<0.01 (Student *t* test).


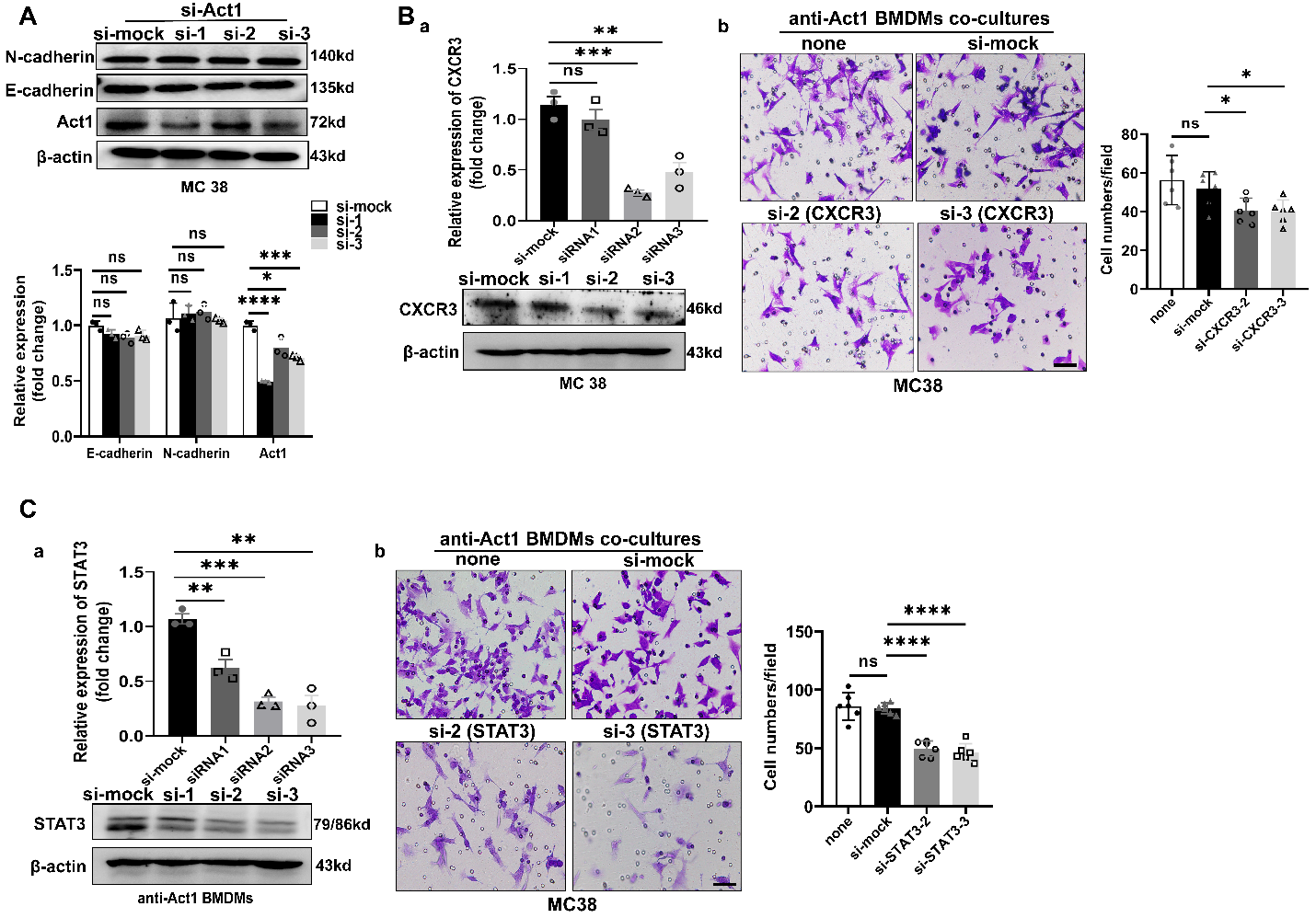


**Fig. S2 The effect of siRNAs on the expression of Act1, CXCR3, and STAT3 in corresponding cells.**

**A.** The effect of siRNAs on Act1 expression in MC38 cells and the effect of Act1 knockdown on EMT markers expression. **B.** The effect of siRNAs on CXCR3 expression in MC38 cells **(a)**; The effect of CXCR3 knockdown in MC38 cells on anti-Act1 macrophages-mediated migration of MC38 cells after coculture 24 h **(b)**; Scale bar, 50 μm. **C.** The effect of siRNAs on STAT3 expression in anti-Act1 BMDMs **(a)**; after coculture 24 h **(b)**. Data are shown as means ± s.e.m. of three independent experiments with at least three independent measurements/experiment. Significant difference between the groups, **p*<0.05, ***p*<0.01, and ****p*<0.001, *****p*<0.0001 (Student *t* test).


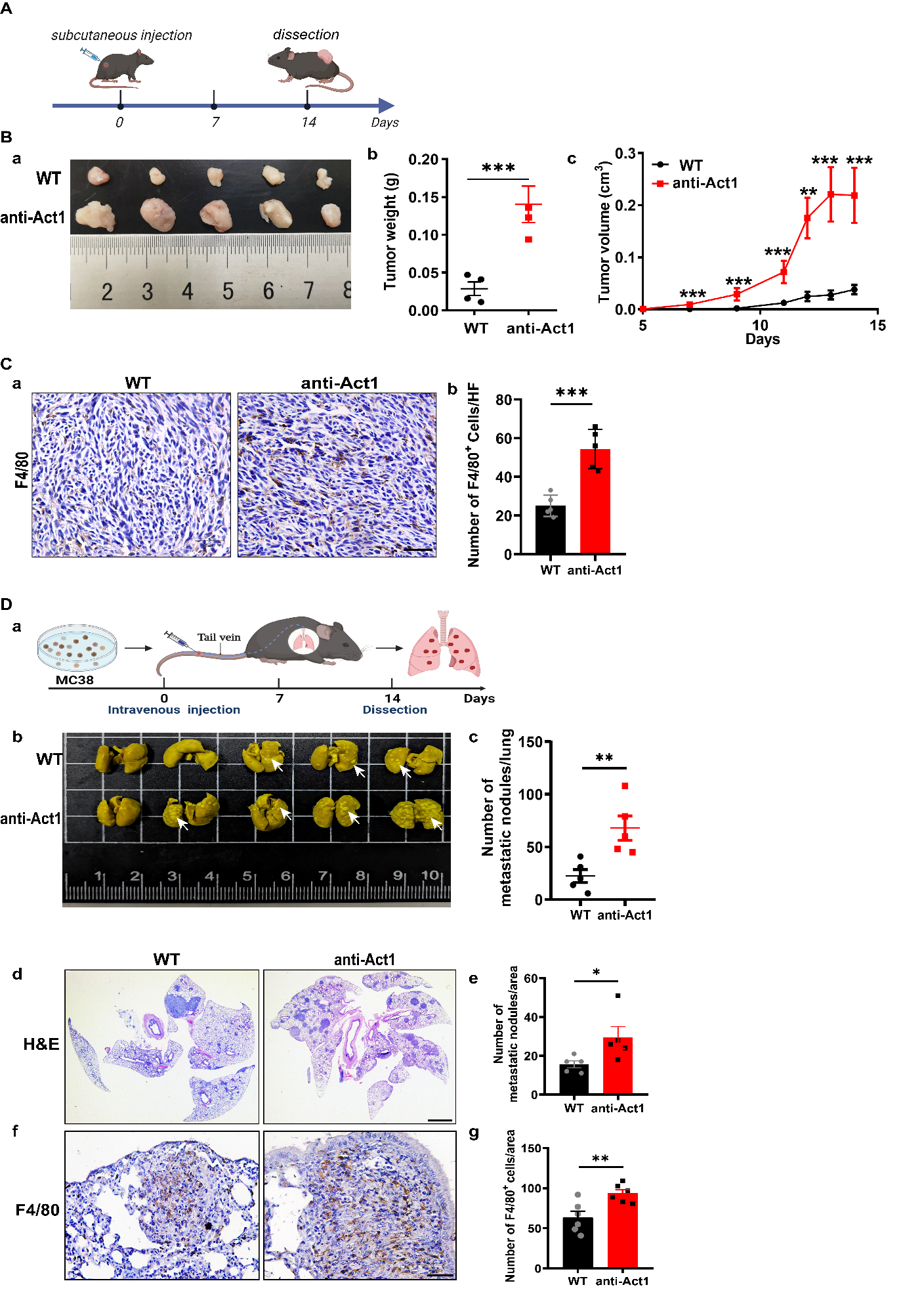


**Fig. S3 The effect of macrophage-specific Act1 knockdown on a xenograft model of CRC cells and lung metastasis mouse model of CRC.** **A.** Graphical depiction of xenograft model; **B**. Representative images of subcutaneous tumors in C57 and anti-Act1 mice **(a)**; Quantitative analysis of tumor weight **(b)**; Quantitative analysis of tumor volume **(c)**; **C.** Immunohistochemical staining **(a)** and quantitative evaluation **(b)** of the number of macrophages in subcutaneous tumors of C57 and anti-Act1 mice. Scale bars, 50 μm. **D**. Graphical depiction of lung metastasis mouse model of CRC **(a)**; Gross lung appearance after fixed by bouin’s fixative **(b);** Quantative statistics of metastasis nodules in the lung **(c)**; H&E staining. Scale bar, 2 mm **(d)**; Statistical analysis of metastatic nodules in lung tissues **(e)**; Immunohistochemical staining for F4/80^+^ macrophages. Scale bar, 100 μm **(f)**; Quantitative evaluation **(g)** of the number of macrophages in lung metastasis model of C57 and anti-Act1 mice. Data are shown as means ± s.e.m. of three independent experiments with at least three independent measurements/experiments. Significant difference between the groups, **p*<0.05, ***p*<0.01, and ****p*<0.001 (Student *t* test).

**
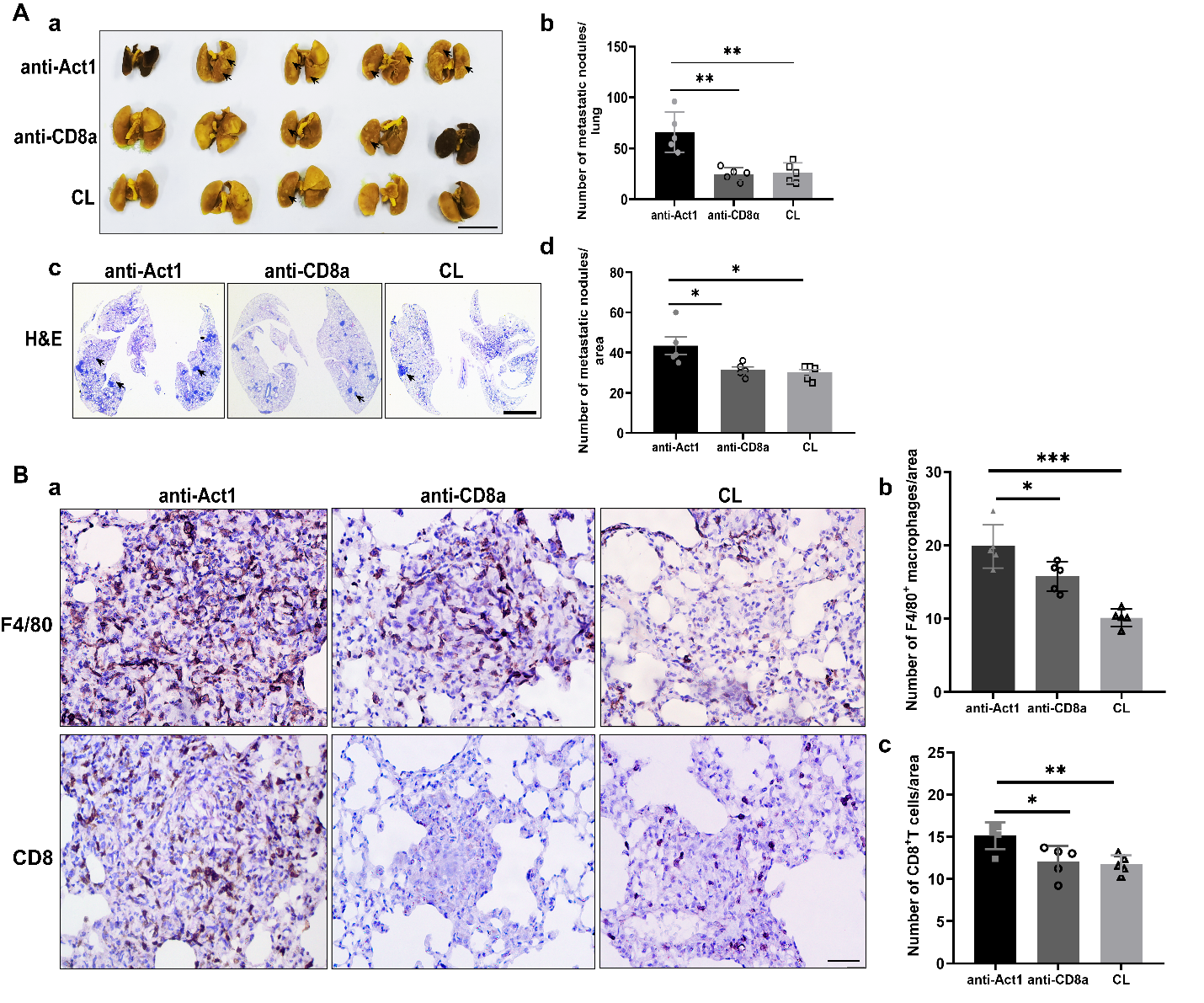
**

**Fig. S4 The effect of macrophage depletion or anti-CD8a therapy in the lung metastasis mouse model of CRC two weeks after being administrated with MC38 cells.**

**A.** Gross lung appearance after fixed by bouin’s fixative **(a)**; The numbers of metastasis nodules on the surface of lung. Scale bar, 1cm. **(b)**; H&E (hematoxylin and eosin) staining**.** Scale bar, 2 mm **(c)**; The numbers of metastasis nodules in the lung tissues **(d)**. **B.** The number of F4/80^+^ macrophages or CD8^+^ T cells in lung tissue was quantified by immunohistochemical staining**. (a)** Representative graph of F4/80^+^ macrophages or CD8^+^ T cells staining by immunohistochemistry. Scale bar, 50 μm. **(b)** Statistic analysis of number of F4/80^+^macrophages; **(c)** Statistic analysis of number of CD8^+^T cells. Data are shown as means ± s.e.m. of three independent experiments with at least three independent measurements/experiment. Significant difference between the groups, **p*<0.05, ***p*<0.01, and ****p*<0.001 (Student *t* test). CL: clodronate liposomes.


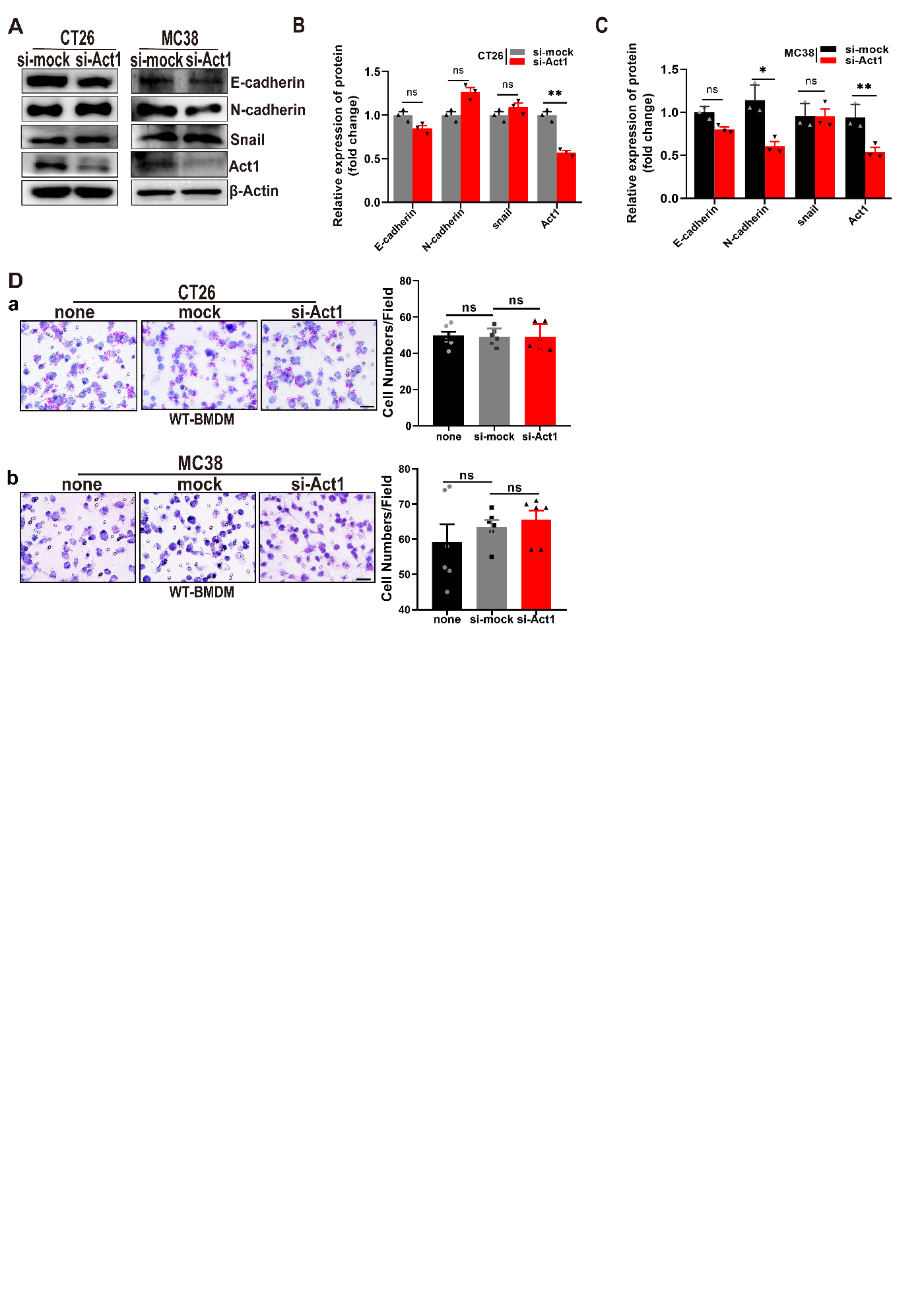


**Fig. S5 The effect of Act1 knockdown in CRC cells on the expression of EMT Markers. A.** Expressions of EMT markers in Act1 knock-downed CRC cells after 48 h detected by immunoblotting. **B.** quantitative assays of EMT markers in CT26 cell line. **C.** quantitative assays of EMT markers in MC38 cell line. **D.** The effect of Act1knockdown in CRC cell lines on the migration of BMDMs after coculture 24h. **(a)** Representative graph and quantitative statistics of BMDMs migration after coculture with CT26 cell lines. **(b)** Representative graph and quantitative statistics of BMDMs migration after coculture with MC38 cell lines. Scale bar, 50 μm. Data are shown as means ± s.e.m. of three independent experiments with at least three independent measurements/experiment. Significant difference between the groups, **p*<0.05 and ***p*<0.01 (Student *t* test).

**
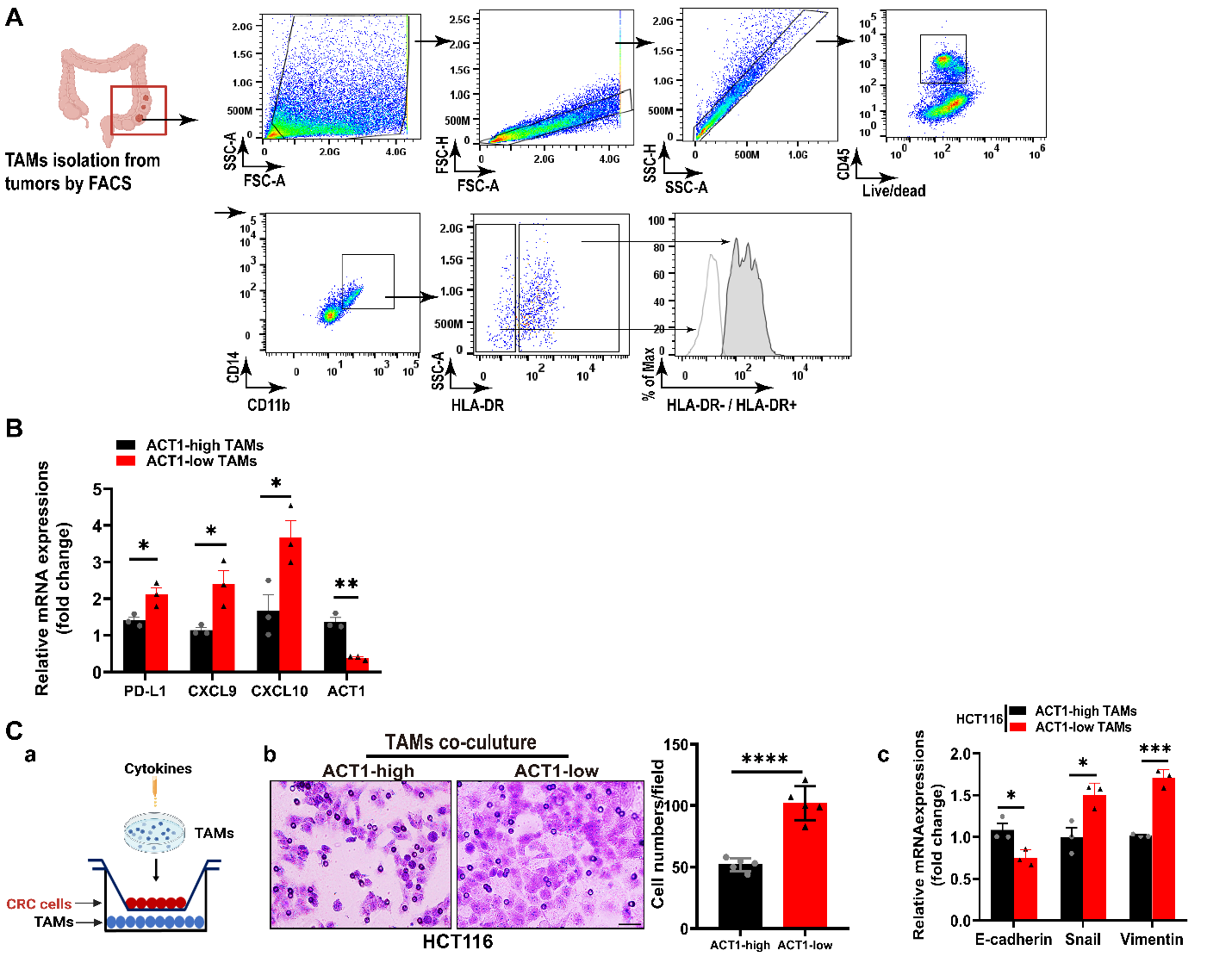
**

**Fig. S6 The effects of TAMs from CRC patients on the migration and EMT of CRC cell line. A.** Schematic graph and representative gating strategy for the identification and collection of TAMs from tumor tissues of CRC patients. Using a gate based on physical parameters (size and granularity) and removing doublet, cells that viable and CD45 positive are selected. Then cells that are CD11b^+^ CD14^+^ HLA-DR^+^ (*grey-lined empty histogram= CD11b^+^ CD14^+^ HLA-DR^-^ macrophages, light grey-colored histogram= macrophages (TAMs) from CRC tumor*) are defined and collected as TAMs in tumor tissues**. B.** Relative mRNA expressions in ACT1-high TAMs (Ct value < 30) and ACT1-low TAMs (Ct value ≥30) isolated from CRC patients. **C.** Schematic graph of coculture between TAMs and human CRC cell line **(a)**; Representative photos and quantitative statistics of HCT116 cell lines migration after coculture with TAMs 48 h. Scale bar, 50 μm (**b)**; Relative mRNA expression of EMT markers in HCT116 cells after coculture with TAMs 48 h. **(c)**. Data are shown as means ± s.e.m. of three independent experiments with at least three independent measurements/experiment. Significant difference between the groups, **p*<0.05, ***p*<0.01, and ****p*<0.001(Student *t* test).

**
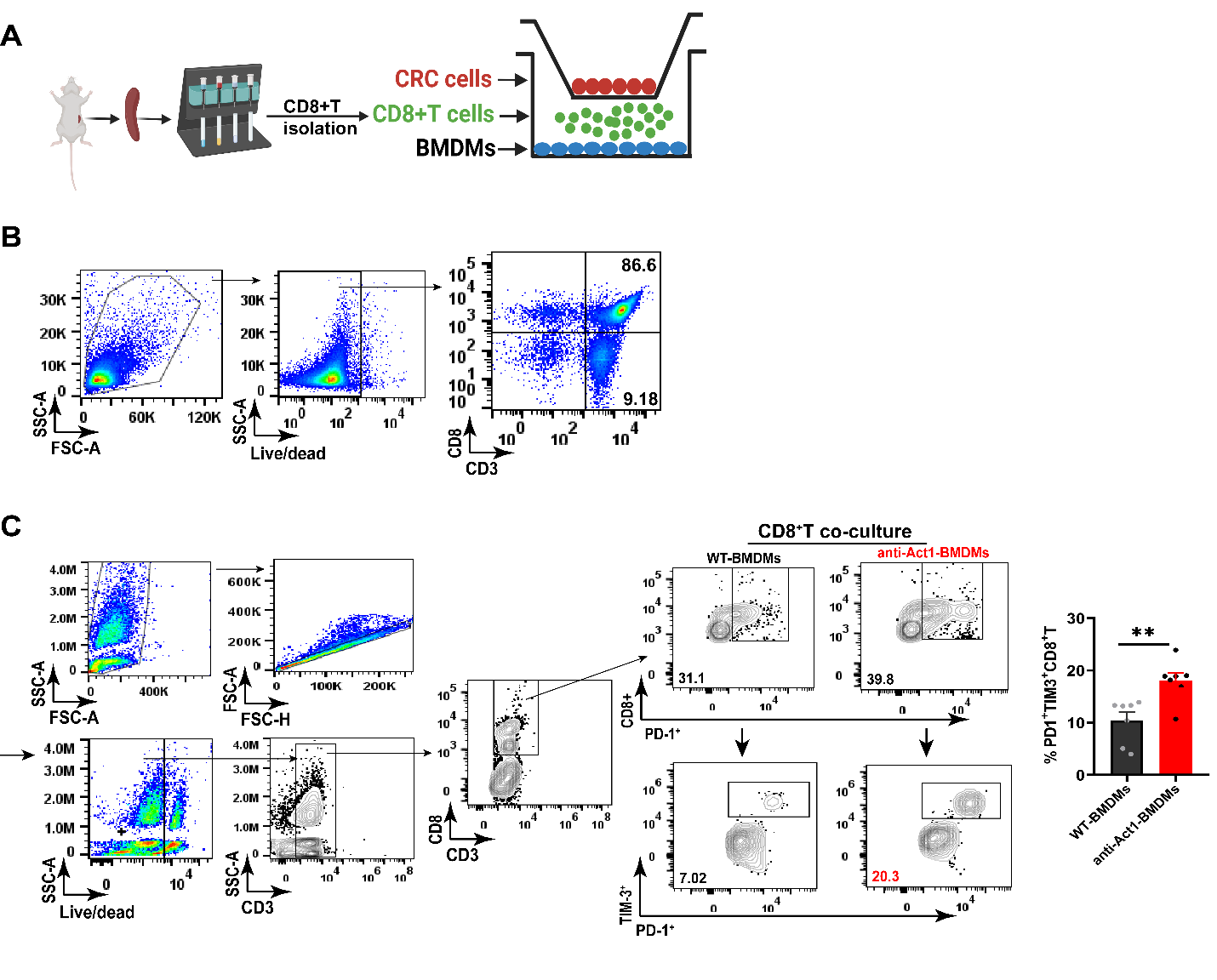
**

**Fig. S7 The effect of Act1-knockdown in BMDMs on the CD8^+^ T cells exhaustion.**

**A.** Schematic graph of the experimental design; **B.** Purity of CD8^+^ T cell enrichment by magnetic bead; **C.** Identification and quantitative statistic of exhaustive CD8^+^ T cell in coculture system. Data are shown as means ± s.e.m. of three independent experiments with at least three independent measurements/experiment. Significant difference between the groups, ***p*<0.01 (Student *t* test).
