# Supplementary material for: NF-κB Activator 1 downregulation in macrophages activates STAT3 to promote adenoma-adenocarcinoma transition and immunosuppression in colorectal cancer
Source: BMC Med. 2023 Mar 29;21:115. doi: 10.1186/s12916-023-02791-0 (PMC10053426; doi:10.1186/s12916-023-02791-0)
Supplement: Supplementary file 3 — Additional file 3. Supplementarymethods. [file 12916_2023_2791_MOESM3_ESM.docx]

**NF-κB Activator 1 Downregulation in Macrophages Activates STAT3 to Promote Adenoma-adenocarcinoma Transition and Immunosuppression in Colorectal Cancer**

Shunyi Wang^1#^, Yihe Kuai^1#^, Simin Lin^2 #^, Li Li^1#^, Quliang Gu^1^, Xiaohan Zhang^3^, Xiaoming Li^4^, Yajun He^4^, Sishuo Chen^1^, Xiaoru Xia^1^, Zhang Ruan^1^, Caixia Lin^1^, Yi Ding^1^, Qianqian Zhang^1^, Cuiling Qi^1^, Jiangchao Li^1^, Xiaodong He^1^, Janak L Pathak^5^, Weijie Zhou^6^, Side Liu^2*^, Lijing Wang^1*^, Lingyun Zheng^1,7*^

**Additional file 3**

Supplementary methods for additional file 2: Fig. S3, S4, S6, and S7.

**Lung metastasis mouse model**

To establish a colon cancer cell lung metastasis model, wildtype mice, and anti-Act1 mice were injected with the MC38 cells (3×10^5^/200 μL phosphate-buffered saline (PBS)) via the tail vein, for an additional 2 weeks.

**Histology and immunohistochemistry**

The lung tissues were fixed in Bouin's solution, paraffin-embedded, sectioned, and stained with H&E. The images were taken with Leica M125C Stereomicroscope (Leica Microsystems, Wetzlar, Germany) under 0.8× field. For immunohistochemistry, 3 µm thick tissue sections were subsequently dewaxed, deparaffinized, and rehydrated, and endogenous peroxidase was quenched with 3% H_2_O_2_ in methanol for 30 min. The slides were blocked with 10% bovine serum albumin (Sigma-Aldrich) at 37 °C for 50 min and then incubated with anti-F4/80 (1:200; #70076s, CST, America) and anti-CD8 (1:200; #ab217344, Abcam, America) antibodies were incubated overnight at 4 °C. Next, the tissue sections were incubated with a horseradish peroxidase-conjugated goat anti-rabbit or anti-mouse IgG antibody (ZSGB-BIO, Beijing, China) at 37 °C for 50 min. The slides were stained with diaminobenzidine solution (Dako Cytomation, Hamburg, Germany), and the cell nuclei were counterstained with hematoxylin. The images were taken with a microscope (OLYMPUS, Japan) 20× field or 40× field. The immunohistochemical staining data were collected and assessed quantified using Image-Pro Plus 4.5 software (Media Cybernetics, MD, USA).by two researchers using a double-blind protocol.

**Clinical samples for TAMs isolation**

We collected 7 colorectal cancer fresh tissue from 7 patients, which were identified by the pathologist of Southern Hospital as "Colorectal cancer" samples. Samples were taken from the middle site of the tumor that was not necrotic and/or hematoma. Fresh colorectal cancer tissue was used for TAMs sorting and colorectal cancer tissue for IHC. The use of clinical samples from colorectal patients was reviewed and approved by the ethics committee of Southern Hospital, and all clinical samples were taken with the informed consent of patients and their families. These colorectal patients’ demographics and clinical information are provided in **Additional file 1: Table S3**.

**TAMs sorting**

Fresh tumor tissue (100–200 mg) was digested into a gentleMACS C Tube (Miltenyi Biotec; cat:130-093-237) containing Collagenase D (Roche; cat:11088858001), DNase I (Sigma-Aldrich; cat: 10104159001), Dispase Ⅱ (Sigma-Aldrich; cat: D4693), and 10 ml RPMI 1640. The C tube was processed on a gentle MACS Octo Dissociator with Heaters (Miltenyi Biotec; cat: 130-096-427) using the program “37C_h_TDK_1” for 45 min. Then, tumor cell suspensions were poured through a 70 μm pre-coated with 2% BSA/PBS. After washing and centrifugation, then 10 min blocking, extracellular staining was performed. Following antibodies against surface epitopes were used: anti-CD45-BV421 (1:100; BD science; clone: HI30), anti-CD11b-PE (1:100; eBioscience; clone: ICRF44), anti-CD14-Percp (1:100; Biolegend; clone: HCD14), anti-HLA-DR-FITC (1:100; eBioscience; clone: LN3), anti-CD3-eFluor 660 (1:100; eBioscience; clone: OKT3), anti-CD19-APC (1:100; MULTISCIENCES; clone: HIB19), anti-CD56-APC (1:100, eBioscience; clone: TULY56), and Zombie NIR™ Fixable Viability Kit (BioLegend; cat: 423101), then sorted using MoFlo XDP (Beckman Coulter, USA). FACS analysis was done using FlowJo software (FlowJo, LLC). and the positive staining was identified by comparison to appropriate isotype control to correct for non-specific binding. Sorting gates were established based on the level of marker expression as well as the exclusion of dead cells stained with Fixable Viability Dye and the exclusion of cells exhibiting non-specific binding or autofluorescence (gating strategy are shown in **Additional file 2: Fig. S6**).

**CD8^+^ T cell isolation and enrichment**

Wildtype mouse spleens were collected and dissociated mechanically. Then, CD8^+^ T cells were sorted on a MACS column (Miltenyi Biotec; cat: 130-042-401) by CD8a^+^ T Cell Isolation Kit (Miltenyi Biotec; cat:130-104-075). All procedures followed the manufacturer's protocol. Subtypes of collected CD8^+^ T cells were confirmed using flow cytometry (**Additional file 2: Fig. S7; routine purity >85%**).

**BMDMs, CD8^+^ T cells, and CRC cell lines coculture**

The coculture systems were established through 6-well plates and 0.4 μm pore size transwell inserts (Corning)^24^. Before coculture, 6-well plates were coated with anti-CD3e (BD biosciences, clone:145-2C11) and anti-CD28 (BD biosciences, clone:37.51) at 37℃ incubators for 3 h. BMDMs (1×10^6^ cells/well) were seeded in 6-well plates and MC38 CRC cells (5×10^5^/insert) were seeded in transwell inserts. CD8+T cells were directly added to BMDMs at a concentration of 5×10^5^ cells/ml.

**Flow cytometry analysis**

Coculture CD8^+^ T cells were analyzed using FACSCelesta flow cytometer (BD Biosciences). Cells were first stained with anti-CD16/32 (Biolegend; clone:93) for 10 min at 4 C, then with specifically conjugated antibodies for 1 h at 4 C in the dark. The following anti-mouse antibodies were used in the analysis: anti-CD3-PerCP/Cyanine5.5 (1:100; Biolegend; clone:17A2), anti-CD8-APC (1:100; Biolegend; clone: 53-6.7), anti-PD-1- FITC (1:100; eBioscience; clone: RMPI-30), anti-Tim3-super Bright 600 (1:100; eBioscience; clone: 8B.2C12) and Fixable Viability Dye (eBioscience; cat: 65-0868).
