# Supplementary material for: NF-κB Activator 1 downregulation in macrophages activates STAT3 to promote adenoma-adenocarcinoma transition and immunosuppression in colorectal cancer
Source: BMC Med. 2023 Mar 29;21:115. doi: 10.1186/s12916-023-02791-0 (PMC10053426; doi:10.1186/s12916-023-02791-0)

**NF-κB Activator 1 Downregulation in Macrophages Activates STAT3 to Promote Adenoma-adenocarcinoma Transition and Immunosuppression in Colorectal Cancer**

Shunyi Wang^1#^, Yihe Kuai^1#^, Simin Lin^2 #^, Li Li^1#^, Quliang Gu^1^, Xiaohan Zhang^3^, Xiaoming Li^4^, Yajun He^4^, Sishuo Chen^1^, Xiaoru Xia^1^, Zhang Ruan^1^, Caixia Lin^1^, Yi Ding^1^, Qianqian Zhang^1^, Cuiling Qi^1^, Jiangchao Li^1^, Xiaodong He^1^, Janak L Pathak^5^, Weijie Zhou^6^, Side Liu^2*^, Lijing Wang^1*^, Lingyun Zheng^1,7*^

**Additional file 5: Images of original blots**


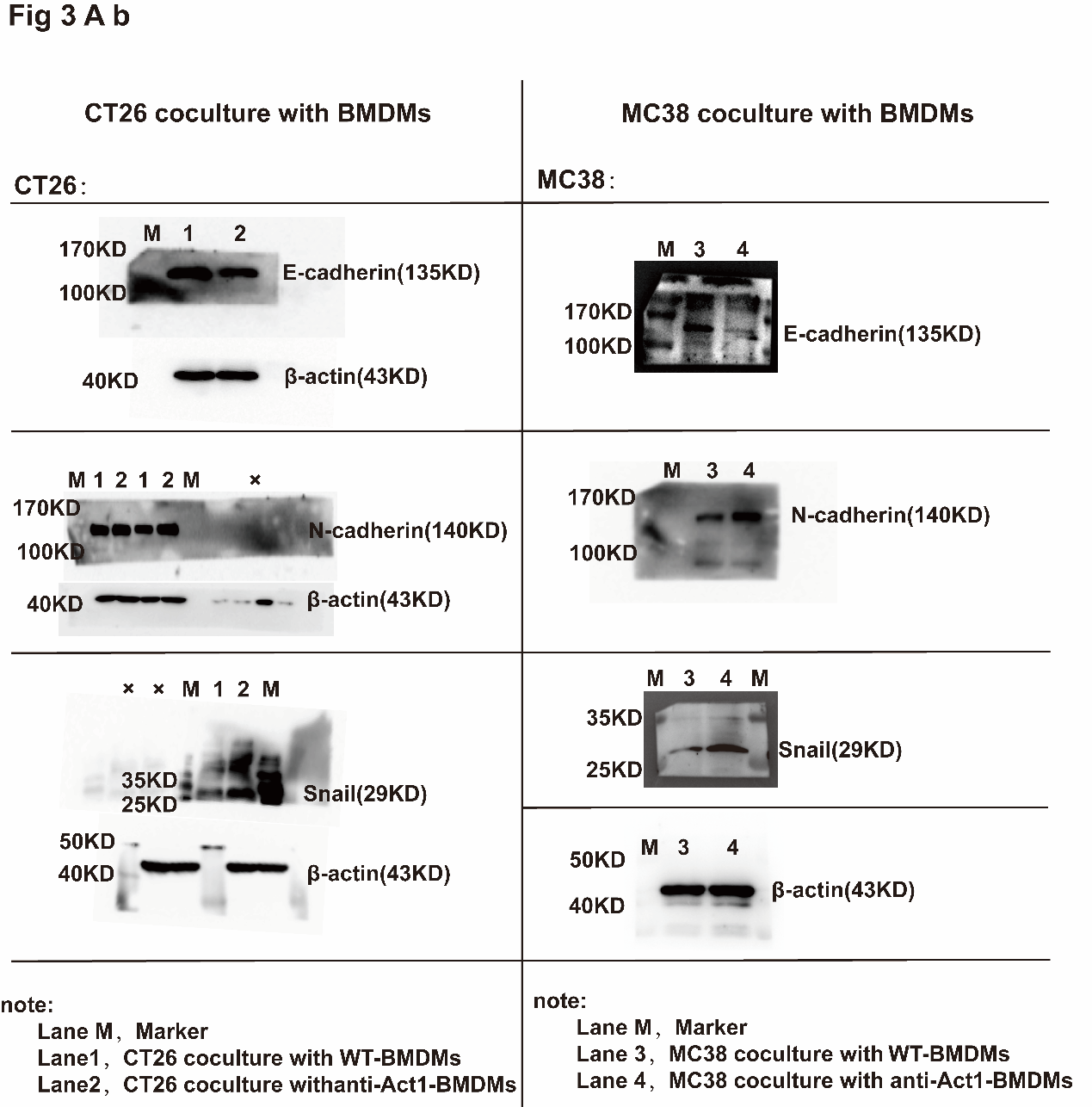


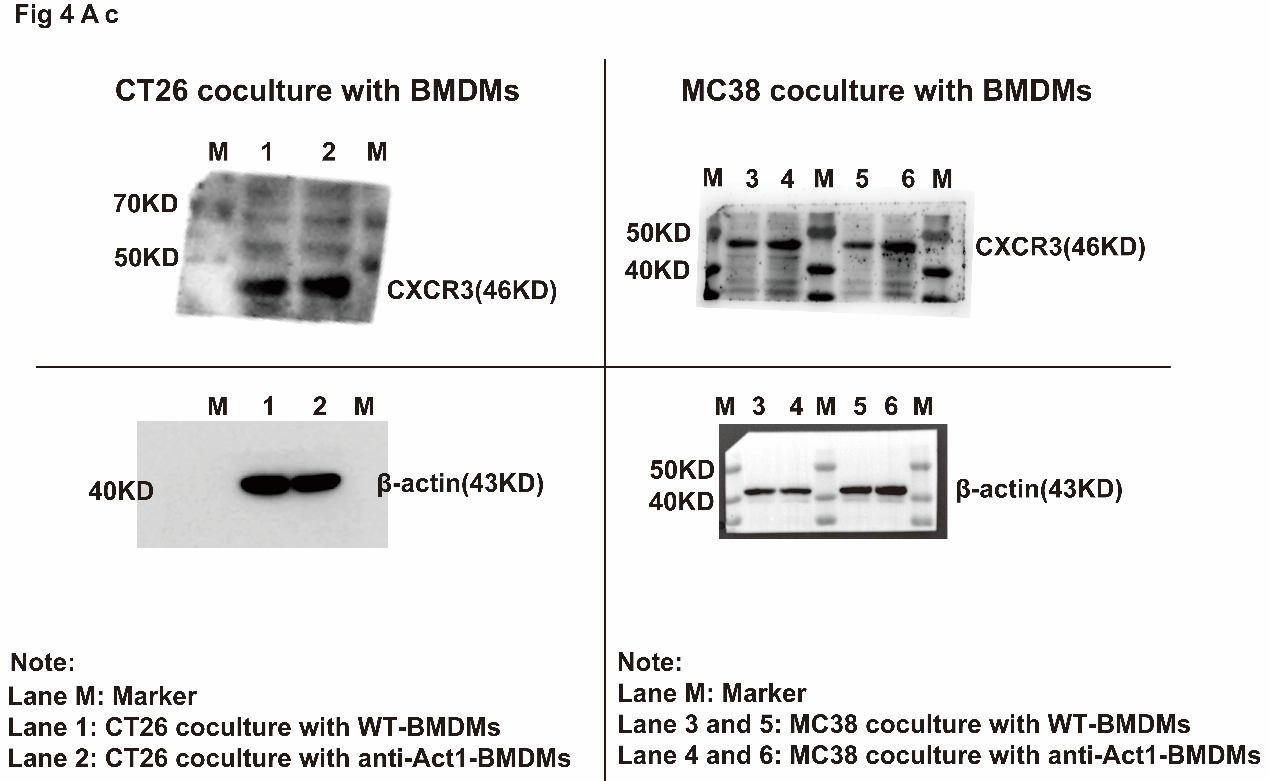


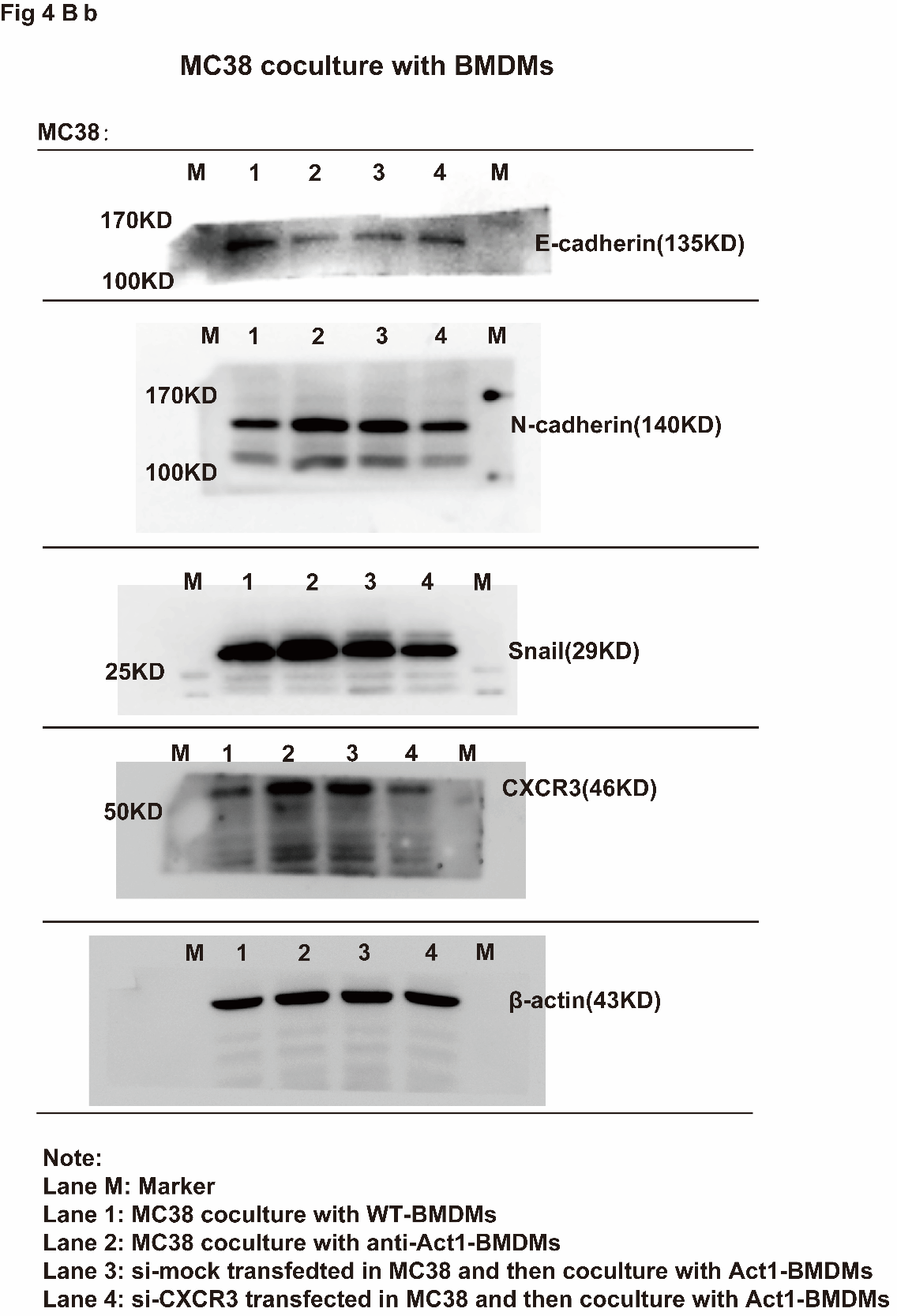


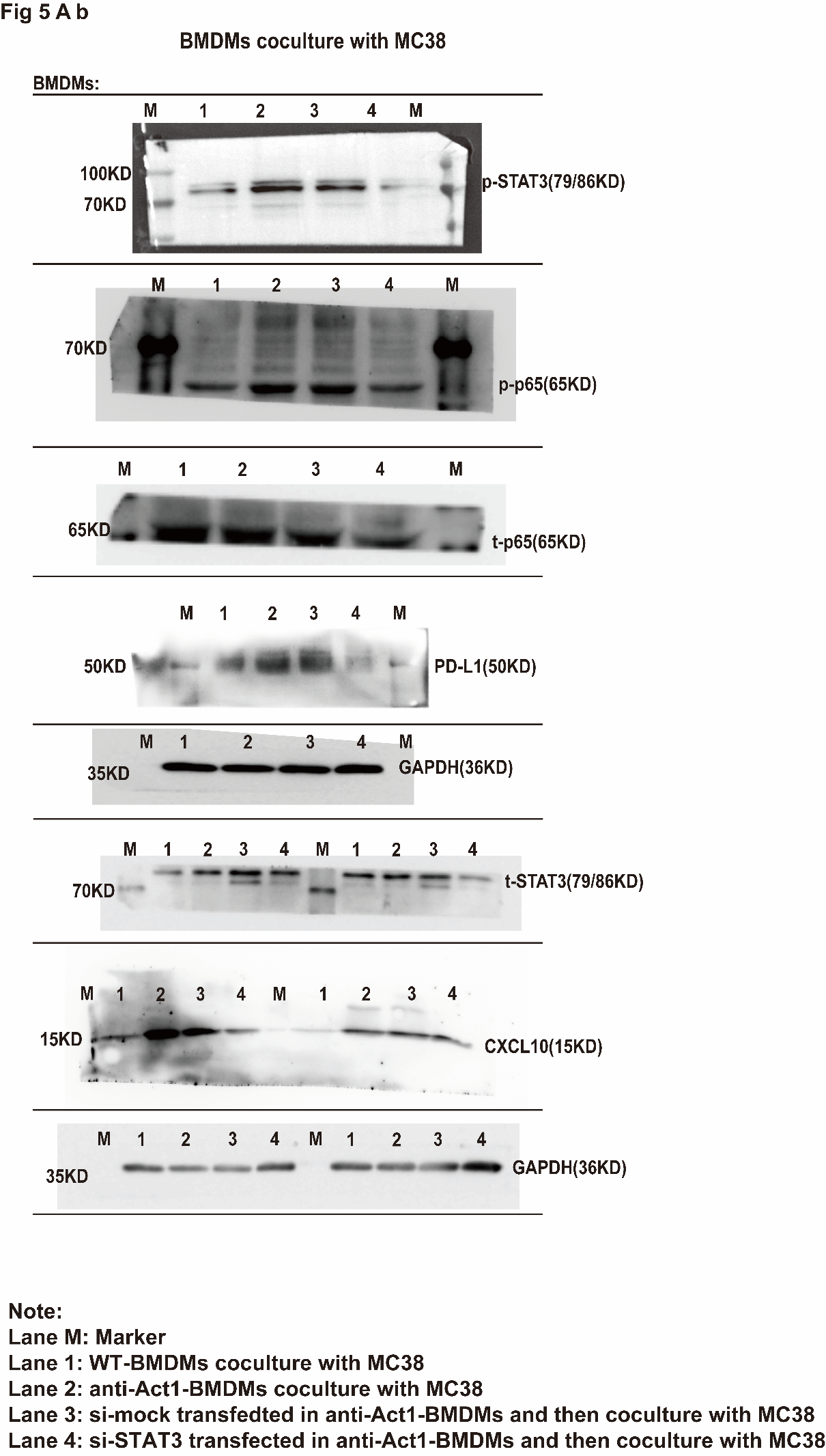


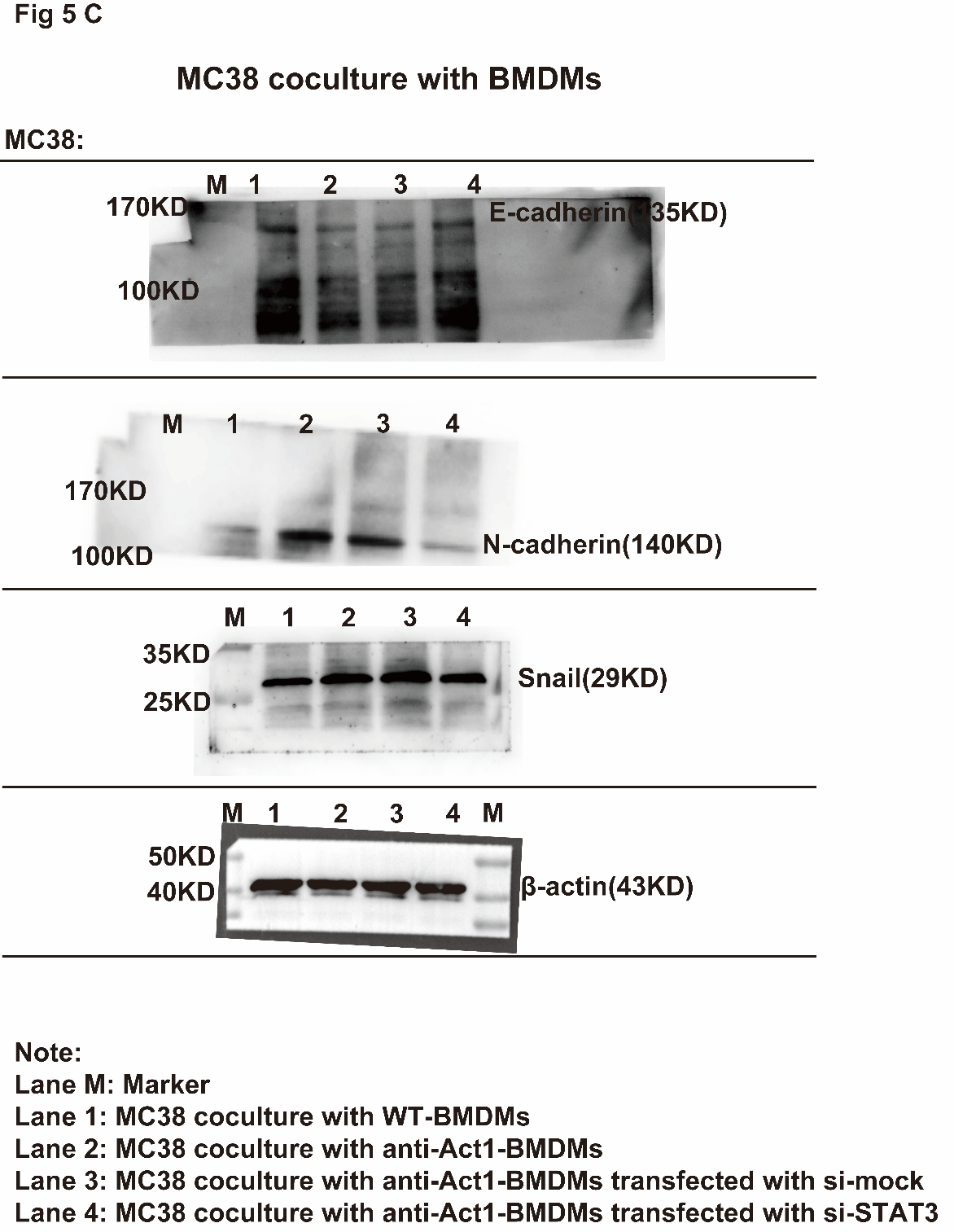


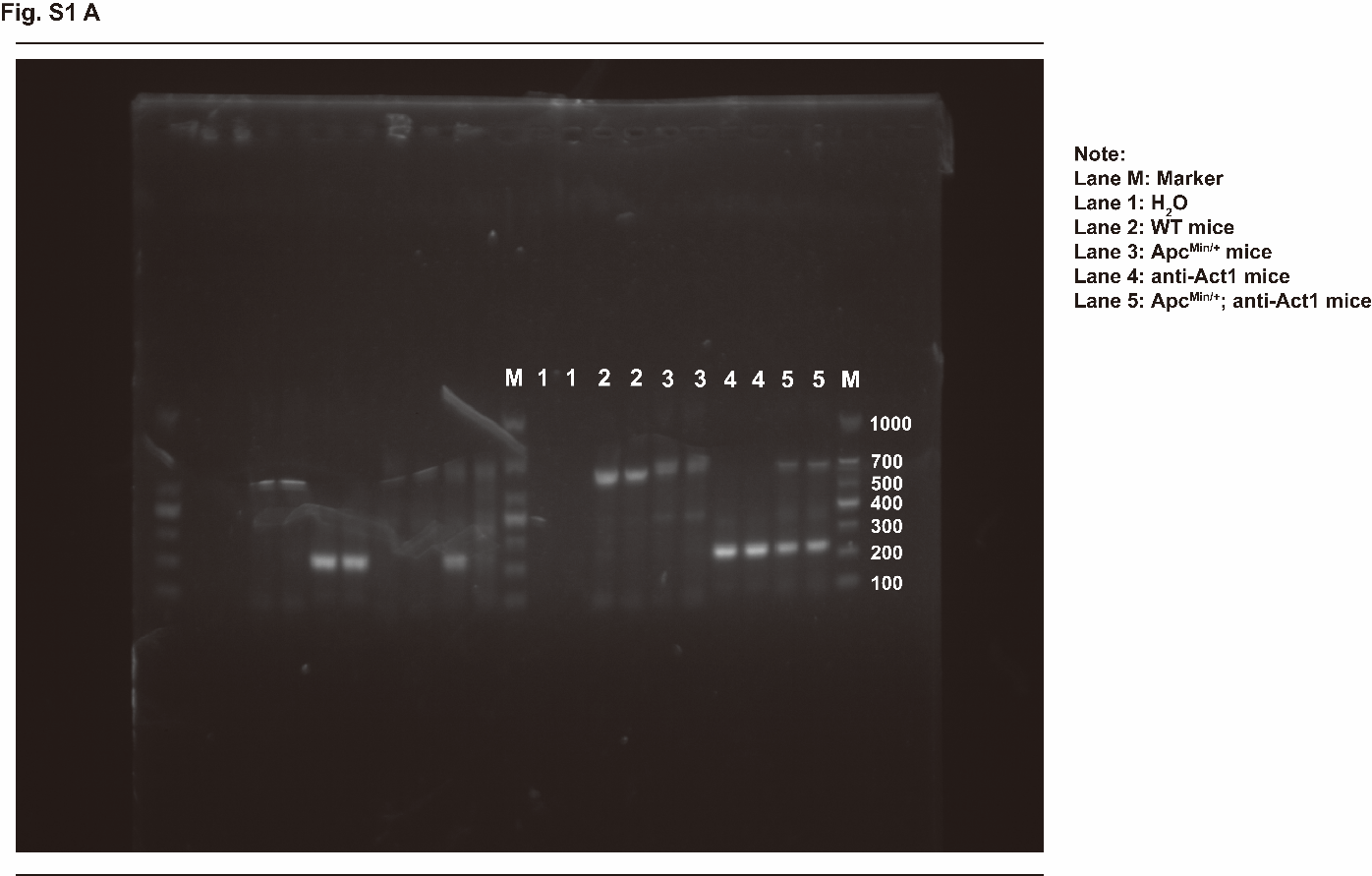


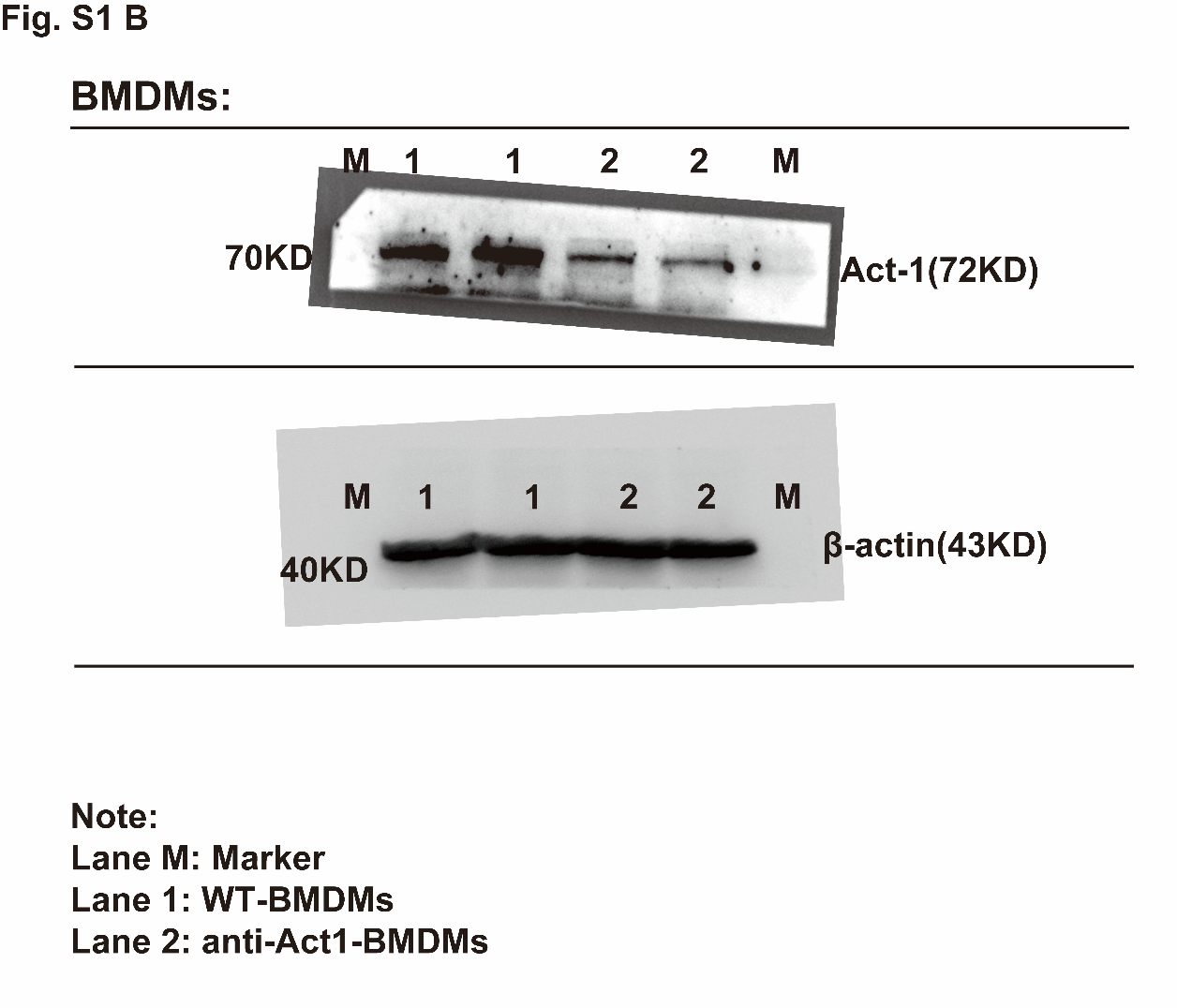


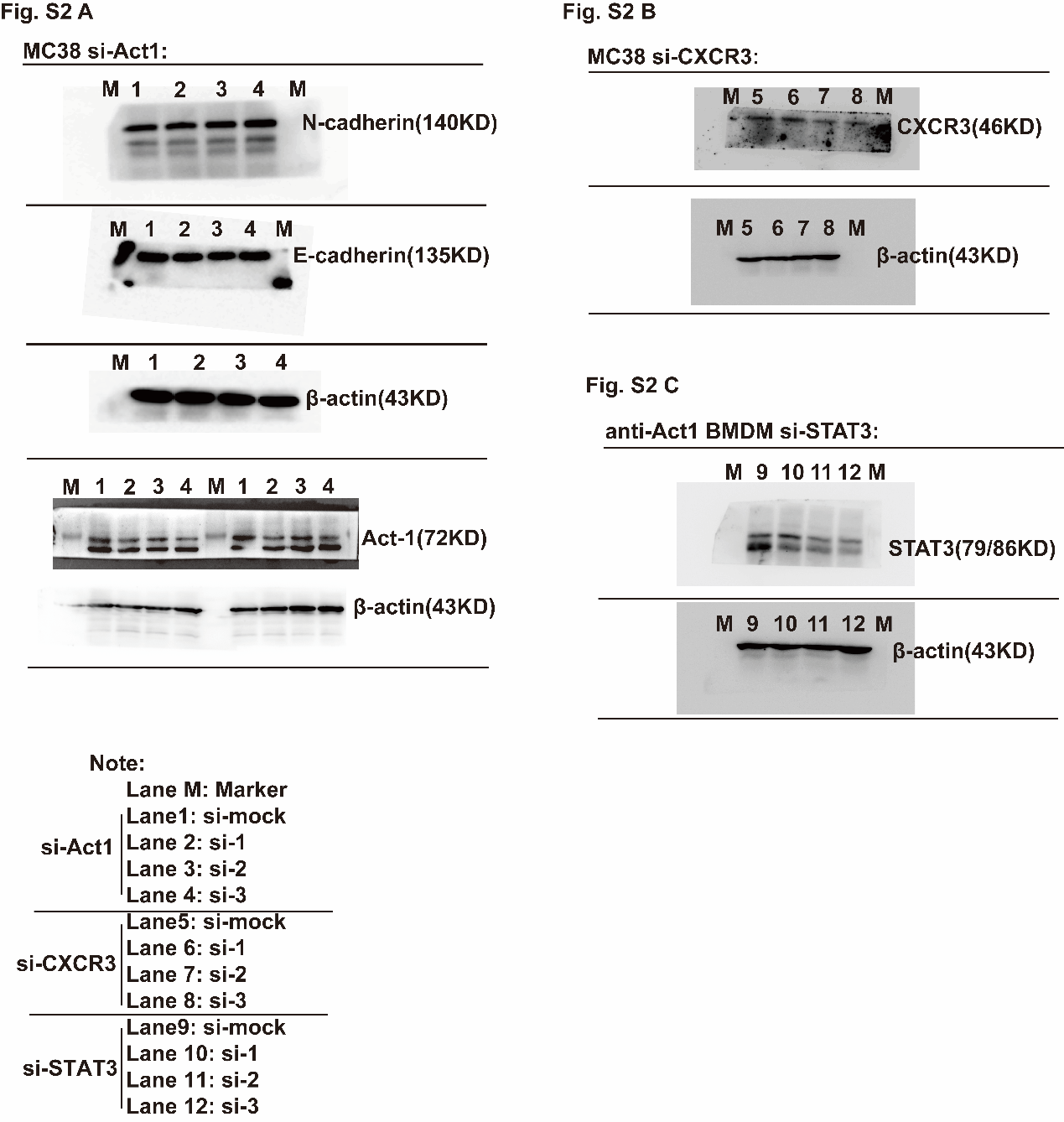


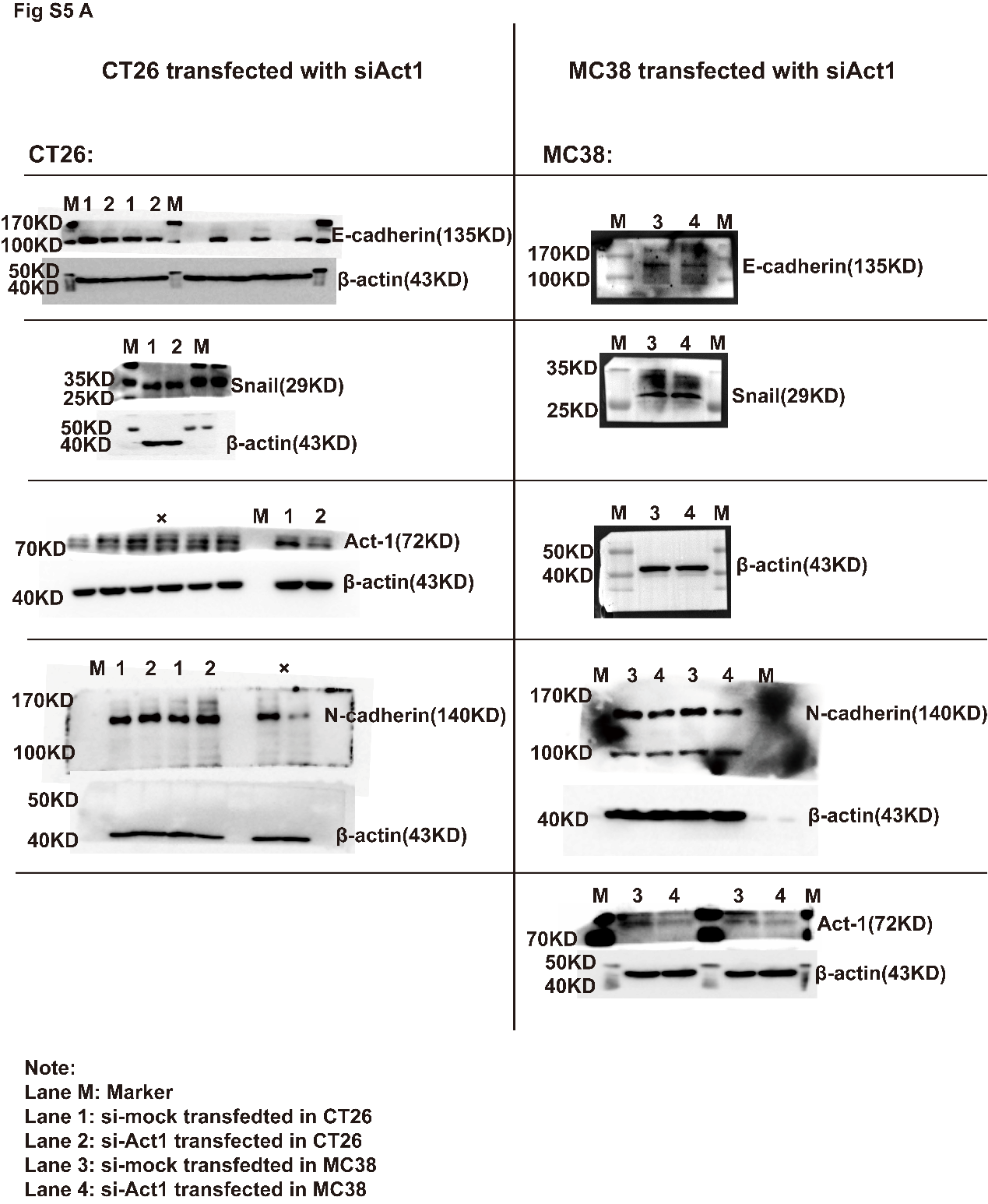

Supplement: Supplementary file 5 — Additional file 5. Imagesof the original blot. [file 12916_2023_2791_MOESM5_ESM.docx]
